# Genome-Wide Identification of BAHD Acyltransferases and *In vivo* Characterization of HQT-like Enzymes Involved in Caffeoylquinic Acid Synthesis in Globe Artichoke

**DOI:** 10.3389/fpls.2016.01424

**Published:** 2016-09-23

**Authors:** Andrea Moglia, Alberto Acquadro, Kaouthar Eljounaidi, Anna M. Milani, Cecilia Cagliero, Patrizia Rubiolo, Andrea Genre, Katarina Cankar, Jules Beekwilder, Cinzia Comino

**Affiliations:** ^1^Department of Agricultural, Forest and Food Sciences, University of TorinoGrugliasco, Italy; ^2^Department of Drug Science and Technology, University of TorinoTorino, Italy; ^3^Department of Life Sciences and Systems Biology, University of TorinoTorino, Italy; ^4^Plant Research InternationalWageningen, Netherlands

**Keywords:** *Cynara cardunculus*, caffeoylquinic acids, BAHD acyltransferases, functional characterization, VIGS

## Abstract

Globe artichoke (*Cynara cardunculus* L. var. *scolymus*) is a rich source of compounds promoting human health (phytonutrients), among them caffeoylquinic acids (CQAs), mainly represented by chlorogenic acid (CGA), and dicaffeoylquinic acids (diCQAs). The enzymes involved in their biosynthesis belong to the large family of BAHD acyltransferases. Following a survey of the globe artichoke genome, we identified 69 BAHD proteins carrying the catalytic site (HXXXD). Their phylogenetic analysis together with another 43 proteins, from 21 species, representative of the BAHD family, highlighted their grouping in seven major clades. Nine globe artichoke acyltransferases clustered in a sub-group of Clade V, with 3 belonging to hydroxycinnamoyl-CoA:quinate hydroxycinnamoyl transferase (HQT) and 2 to hydroxycinnamoyl-CoA:shikimate/quinate hydroxycinnamoyl transferase (HCT) like proteins. We focused our attention on the former, HQT1, HQT2, and HQT3, as they are known to play a key role in CGA biosynthesis. The expression of genes coding for the three HQTs and correlation of expression with the CQA content is reported for different globe artichoke tissues. For the first time in the globe artichoke, we developed and applied the virus-induced gene silencing approach with the goal of assessing *in vivo* the effect of HQT1 silencing, which resulted in a marked reduction of both CGA and diCQAs. On the other hand, when the role of the three HQTs was assessed in leaves of *Nicotiana benthamiana* through their transient overexpression, significant increases in mono- and diCQAs content were observed. Using transient GFP fusion proteins expressed in *N. benthamiana* leaves we also established the sub-cellular localization of these three enzymes.

## Introduction

Plant phenolics, and in particular the caffeoylquinic acids (CQAs), can synergistically or additively provide protection against damage induced by free radicals during oxidative stress, and reduce the risk of chronic diseases in humans (Arakawa et al., 2009; Puangpraphant et al., 2011; Markovic and Tošovic, 2016). The antioxidant activity of CQAs is influenced by the number and position of attachment of caffeic acid moieties on quinic acid (Wang et al., 2003; Xu et al., 2012), while their bioactivity varies according to their isomerisation, which is significantly affected by the extraction method adopted (Mullen et al., 2011). CQAs also play key roles in increasing plant protection from harmful UV light (Cle et al., 2008) as well as in resistance of plants to bacteria (Niggeweg et al., 2004) and insects (Leiss et al., 2009).

Caffeoylquinic acids are produced as monoesters (monocaffeoylquinic acids, monoCQAs, which include chlorogenic acids, CGA) and diesters [dicaffeoylquinic acids, (diCQAs)] by members of plant families such as Asteraceae (a.k.a Compositae), Solanaceae, and Rubiaceae. In recent years, globe artichoke (*Cynara cardunculus* L. var. *scolymus*), a member of the Asteraceae family, has received renewed interest as a source of bioactive compounds (Lattanzio et al., 2009) due to its high content and diverse spectrum of phenolics. Indeed the edible part of the globe artichoke has been reported to possess the highest total polyphenol content among 29 fresh vegetables under study (Brat et al., 2006) and was ranked first, in antioxidant content, among several selected vegetable crops (Halvorsen et al., 2006). The health-promoting potential of globe artichoke extracts is also supported by many *in vivo* and *in vitro* studies which demonstrate its hepatoprotective (Adzet et al., 1987), anticarcinogenic (Clifford, 2000), antioxidative (Gebhardt, 1997; Brown and Rice-Evans, 1998), antifungal and antibacterial properties (Gebhardt, 2001; Coon and Ernst, 2003; Lattanzio et al., 2009).

**FIGURE 1:**
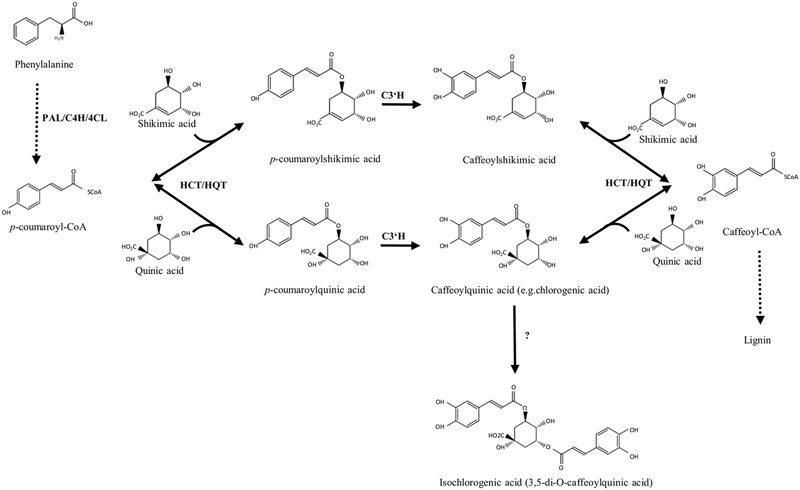
**Biosynthetic pathways of chlorogenic acid and its related derivatives.** PAL, phenylalanine ammonia lyase; C4H, cinnamate 4-hydroxylase; 4CL, 4-hydroxycinnamoyl-CoA ligase; HCT, hydroxycinnamoyl-CoA shikimate/quinate hydroxycinnamoyl transferase; HQT, hydroxycinnamoyl CoA quinate hydroxycinnamoyl transferase; C3’H, *p*-coumaroyl ester 3′-hydroxylase.

The most abundant phenolic acids in globe artichoke heads are CQA esters, mainly CGA (5-*O*-CQA), 1,5-diCQA, and 3,5-diCQA (Lattanzio et al., 1994; Schutz et al., 2004), which are synthesized via the phenylpropanoid pathway (**Figure 1**; Comino et al., 2009). Three routes have been proposed for the synthesis of CGA in plants. In the first, hydroxycinnamoyl-CoA:quinate hydroxycinnamoyl transferase (HQT) catalyzes the formation of CGA from caffeoyl-CoA and quinic acid (Niggeweg et al., 2004; Comino et al., 2009; Menin et al., 2010); the second route is based on the synthesis of *p-*coumaroylquinate by hydroxycinnamoyl-CoA:shikimate hydroxycinnamoyl transferase (HCT), followed by hydroxylation by C3’H (*p*-coumaroyl-3′-hydroxylase; Ulbrich and Zenk, 1979; Hoffmann et al., 2003; Mahesh et al., 2007; Moglia et al., 2009); in the third, caffeoyl glucoside serves as an activated intermediate (Villegas and Kojima, 1986).

However, the biosynthesis of diCQAs is still unclear. In sweet potato the synthesis of isochlorogenate (3,5-di-*O*-caffeoylquinate) has been described (Villegas and Kojima, 1986), but the gene encoding the enzyme catalyzing this reaction has not been identified. Recently, the *in vitro* synthesis of diCQAs from CGA and CoA, mediated by a recombinant HCT enzyme cloned from coffee, was reported (Lallemand et al., 2012), while in tomato, the enzyme HQT was shown *in vitro* to convert CGA to diCQAs, whose synthesis likely occurs in the vacuole (Moglia et al., 2014).

In globe artichoke the genes implicated in CGA synthesis, i.e., *HQT* (DQ915589), *HCT* (DQ104740), *Acyltransf_1* (GU248357), *Acyltransf_2* (GU248358), and *C3’H* (FJ225121) have been isolated and characterized (Comino et al., 2007, 2009; Moglia et al., 2009; Menin et al., 2010), but the proof of their functional role *in vivo* has not yet been demonstrated.

Virus-induced gene silencing (VIGS) has been widely used as a plant reverse genetics strategy to analyse gene function (Kumagai et al., 1995; Ruiz et al., 1998) due to its simplicity, robustness and avoidance of the need for stable transformants. However, until now its application to globe artichoke has not been reported. We have applied here, for the first time, the VIGS strategy in globe artichoke, with the goal of investigating the role of key enzymes in regulating the synthesis of CQAs. In particular, following a genome-wide identification of globe artichoke BAHD acyltransferases, we selected three *HQT*-like genes, whose expression was assessed by quantitative PCR (qPCR) in globe artichoke tissues, and we applied the VIGS technique to evaluate the effect of *HQT1* silencing *in vivo*. Furthermore, in *N. benthamiana*, we estimated the effect of over-expression of the three selected HQT-like enzymes in agro-infiltrated plants and established their sub-cellular localization.

## Materials and Methods

### Identification of Putative BAHD Family Members

Published BAHD acyltransferase sequences (D’Auria, 2006; Tuominen et al., 2011) were used in preliminary BlastP searches against the globe artichoke predicted proteome (Scaglione et al., 2016). The putative BAHD sequences found were aligned with previously characterized BAHD proteins using MUSCLE^1^ and a manual inspection was conducted to exclude *loci* lacking the conserved motifs (HXXXD or DFGWG), with filtering for redundancy. Sequences which exhibited no HXXXD motif were removed. Target P (Emanuelsson et al., 2007) and Predotar (Small et al., 2004) software were used to predict *in silico* the occurrence of mitochondrial, plastid, and ER targeting sequences.

### Phylogenetic Analysis

Globe artichoke putative BAHD sequences together with other characterized protein members (Data Sheet 1) belonging to the 8 clade-based classification reported in Tuominen et al. (2011) were aligned using the MAFFT v6.717 online server^2^; the FFT-NS-i iterative refinement method was run with default settings using the Blosum62 substitution matrix, leaving gappy regions. An UPGMA based phylogenetic tree was constructed and visualized with the FigTree graphical viewer^3^.

### CQA-Related BAHD Ohnolog Genes

Paralogous genes are typically generated by a whole genome duplication (WGD) event (Ohno et al., 1968). The CoGe platform^4^ for comparative genomics was used to detect CQA-related paralogous BAHD genes within the globe artichoke genome. To compute chains of syntenic genes found within the complete genome sequence, DAGchainer software (with the ‘Relative gene order’ option activated and the ‘Maximum distance between two matches’ parameter set to 20) was used together with Quota-Align algorithm (with maximum distance between two blocks set to 20 genes), both implemented to the SynMap function within CoGe. The chromosomal locations of the ohnolog BAHD genes were visualized using CIRCOS ideograms generated by the software package from http://circos.ca.

### Quantitative PCR and LC-QTOF-MS Analysis in Globe Artichoke Tissues

Globe artichoke plants (F1 hybrid ‘Concerto,’ Nunhems) were grown up to the production of commercial immature inflorescences (heads) in an experimental field at Carmagnola (Torino). The following plant materials were harvested and stored at –80°C until required: (i) leaves from 6 weeks- and 1 year-old plants; (ii) external bracts of the inflorescence at the commercial stage; (iii) stems of the primary head at the commercial stage of the inflorescence.

RNA was isolated from 100 mg of globe artichoke tissues using Trizol reagent (Invitrogen) according to the manufacturer’s instructions. The total RNA was quantified and controlled for purity using a spectrophotometer and agarose gel electrophoresis. cDNAs were synthesised from 1.0 μg total plant tissue RNA using a *High Capacity RNA-to-cDNA Kit* (Thermofisher) according to the manufacturer’s instructions. For quantification of the levels of *HQT, Acyltransf_1*, and *Acyltransf_2* (hereafter named *HQT1, HQT2*, and *HQT3*, respectively,) gene-expression in different globe artichoke tissues, a qPCR analysis was performed, using the primers reported in Supplementary Table 1. As a housekeeping gene for globe artichoke, actin (amplified with the primer combination ACT-Rt-For and ACT-Rt-Rev, Supplementary Table 1) was chosen for its stability and level of expression, comparable to those of the genes of interest, and whose expression remains stable in all tissues (Menin et al., 2012). 20 μL qPCRs were performed in three biological replicates for each tested tissue in the presence of fluorescent dye (GoTaq^®^ qPCR Master Mix, Promega). PCR reactions were carried out in 48-well optical plates using the iCycler Real-time PCR Detection System (Bio-Rad Laboratories, USA) as described in Menin et al. (2010).

Ground tissue (50 mg) of each plant biological replicate was extracted with 1 mL of 75% methanol containing 0.1% formic acid and sonicated (125 W, 20 kHz) for 15 min. Extracts were then centrifuged at 20,000 g and 22°C for 5 min, filtered through a 0.2 μm inorganic membrane filter (RC4, Sartorius, Germany) attached to a disposable syringe, and transferred to a glass vial. The LC-QTOF-MS platform consisted of a Waters Alliance 2795 HT HPLC system equipped with a Luna C18(2) pre-column (2.0 × 4 mm) and an analytical column (2.0 × 150 mm, pore size 100 Å, particle size 3 μm; Phenomenex), connected to an Ultima V4.00.00 QTOF mass spectrometer (Waters, MS Technologies). Degassed eluent A, ultra-pure water: formic acid (1000:1, v/v), and eluent B, acetonitrile: formic acid (1000:1, v/v) were used at a total flow rate of 0.19 mL min^-1^. The gradient started at 5% B and increased linearly to 75% B over 45 min; afterward the column was washed with 100% B and equilibrated at 5% A for 15 min before the next injection. The injection volume was 5 μL, ionization was performed using an electrospray source, with detection in the positive mode. The identification of CQAs was carried out by comparing retention times and masses with those reported in Menin et al. (2010), using standard cynarin (1,3-dicaffeoylquinic acid) from Carl Roth (Karlsruhe) and chlorogenic acid from Sigma–Aldrich. Mean comparison was conducted using Tukey’s test. All the data were statistically analyzed using SPSS statistical software.

### Virus Induced Gene Silencing in Globe Artichoke

Seeds of the globe artichoke hybrid ‘Concerto’ were germinated for 2 weeks between two layers of wet filter paper; plantlets were then transplanted into pots in a greenhouse and grown in a climate room at 25°C with 60% relative humidity and a 16 h light: 8 h dark photoperiod cycle with light intensity of 300 μmol m^-2^s^-1^. The pTRV1 and pTRV2 vectors described by Liu et al. (2002) were used in this study. Two pTRV2 based constructs were employed: pTRV2-PDS [phytoene desaturase (PDS)] and pTRV2-PDS-HQT1. In order to identify the cDNA sequence of globe artichoke PDS, the cDNA sequence of tomato *PDS* (Liu et al., 2002) was used as query for blast searches in *C. cardunculus* EST database (Scaglione et al., 2012). 423 bp *PDS* fragment was PCR amplified from globe artichoke cDNA using primers with *Eco*RI and *Xho*I restriction sites (PDS-EcoF and PDS-XhoR, Supplementary Table 1). The resulting product was cloned into pTRV2 to form pTRV2-PDS. 400 bp fragment of *HQT1* was PCR amplified from globe artichoke cDNA using primers with *Xho*I and *SmaI* restriction sites (HQT1-XhoF and HQT1-SmaR, Supplementary Table 1) and cloned into pTRV2-PDS vector.

The pTRV2 constructs were transformed into *Agrobacterium tumefaciens* strain C5801. The obtained recombinant *A. tumefaciens* strains were grown at 28°C and 80 rpm for 24 h in 5 mL of LB media containing kanamycin (50 mg L^-1^) and tetracycline (10 mg L^-1^). After an overnight incubation, 500 μL of the cultured cells were added to 25 mL of LB broth containing 10 mM 2-[N-morpholino] ethanesulfonic acid (MES) and 20 μM acetosyringone (4′-hydroxy-3′,5′-dimethoxyacetophenone, Sigma) and grown overnight at 28°C and 80 rpm. After the overnight incubation, the bacterial cultures were centrifuged for 20 min at 4,000 g and 4°C, resuspended in 10 mM MES buffer containing 10 mM MgCl_2_ and 200 μM acetosyringone to a final OD_600_ of 1–1.5, and incubated at room temperature under gentle shaking at 50 rpm for 3 h. The bacteria containing pTRV1 and the bacteria containing pTRV2 or its derivatives were then mixed together in 1:1 ratio. The cotyledons of globe artichoke were infiltrated with the mixed bacteria cultures using a 1 mL disposable syringe without a needle. The agroinfiltrated plants were then transferred to a climate room at 25°C with 60% relative humidity and a 16 h light/8 h dark photoperiod cycle with light intensity ranging from 300 to 400 μM m^-2^s^-1^. After 4 weeks the VIGS-silenced plant material (3 biological replicates) was collected and used for qPCR (as described in Quantitative PCR and LC-QTOF-MS Analysis in Globe Artichoke Tissues) and LC-PDA analyses. For the quantification of PDS gene-expression levels in silenced material we used primers reported in Supplementary Table 1.

### Transient Heterologous Expression in *Nicotiana benthamiana*

For transient expression, the pEAQ-HT vector (extremely high-level expression, GATEWAY-compatible plasmid, Sainsbury et al., 2009) was kindly provided by Prof. Lomonossoff (JIC, Norwich UK). For the construction of the expression vectors containing HQT1, HQT2, and HQT3, sets of primers with attB1 and attB2 sites (Supplementary Table 1) were designed. The amplified fragments were first cloned by Gateway Recombinant Technology in pDONOR 207 vector through a BP recombination and subsequently transferred by LR recombination into the pEAQ-HT destination vector, originating the expression vectors pEAQ/HQT1, pEAQ/HQT2, and pEAQ/HQT3. These vectors and the empty vector pEAQ-HT, as a negative control, were introduced into *Agrobacterium tumefaciens* strain C5801 by the freeze-thaw method. Bacteria containing a single construct or the control vector were grown overnight at 28°C in 5 mL of L medium (10 g L^-1^ bactotryptone, 5 g L^-1^ Yeast extract, 5 g L^-1^ NaCl, 1 g L^-1^ D-glucose) with kanamycin (50 mg L^-1^). The overnight cultures (2 mL) were then transferred into 20 mL of induction medium (L broth containing 10 mM MES and 20 μM acetosyringone) with kanamycin (50 mg L^-1^), and grown as above. The cells were collected by centrifugation for 10 min at 4,000 g and resuspended in 50 mL of infiltration medium (10 mM MgCl_2_, 10 mM MES, 200 μM acetosyringone) to an OD_600_ of 1.0 and kept at room temperature for 3 h before being infiltrated into the abaxial air spaces of 2–4-week-old *N. benthamiana* plants. After 4 days, the infiltrated leaf material was collected and used for semi-qPCR and LC-QTOF-MS analyses.

For the quantification of transgene expression in transformed *N. benthamiana* plants a semi-qPCR analysis was performed. cDNAs were synthesized from 1.0 μg total RNA from leaves of HQT1, HQT2, HQT3 and control transformants using a High Capacity RNA-to-cDNA Kit (Thermofisher) according to the manufacturer’s instructions. Semi-qPCR amplifications were performed by using specific primers designed by Primer 3 software^5^ for globe artichoke *HQT1, HQT2*, and *HQT3* and tobacco elongation (EF) factor as a housekeeping gene (Nt-EF-For and Nt-EF-Rev, Supplemental Table 1). The thermal cycling program included one step at 95°C for 5 min, followed by 25 cycles of three steps (94°C for 30 s, 57°C for 30 s and 72°C for 30 s). Amplified products were visualized on a 1.5% agarose gel.

### Identification and Quantification of CQAs in Transiently Transformed *N. benthamiana* and in VIGS Silenced Globe Artichoke Tissues

Transiently transformed *N. benthamiana* grinded tissues (100 mg) were suspended in 300 μl of 70% (v/v) methanol and sonicated for 20 min in a water bath. After centrifugation (10,000 g for 10 min), supernatants were filtered with a 13 mm diameter, 0.22 μm pore diameter PTFE syringe filter and analyzed on the LC-PDA-MS/MS analytical platform. Analyses were carried out on a Shimadzu Nexera X2 system equipped with a photodiode detector SPD-M20A in series to a triple quadrupole Shimadzu LCMS-8040 system provided with electrospray ionization (ESI) source (Shimadzu, Dusseldorf Germany). An Ascentis^®^ Express RP-Amide column (100 mm × 2.1 mm i.d., 2.7 μm particle size, Supelco, Bellefonte, PA) was used. The analysis conditions were: mobile phase: eluent A: 0.1% formic acid in water; eluent B: 0.1% formic acid in acetonitrile; mobile phase gradient was as follows: 5–25% B in 20 min, 25–100% B in 10 min, and 100% B for 1 min. Injection volume: 5 μL; the flow rate of the mobile phase was 0.4 mL min^-1^ and the column was maintained at 30°C. UV spectra were acquired in the 210–450 nm wavelength range.

The identification of the components was based on their UV spectra and mass spectral information in Multiple Reaction Monitoring (MRM) mode in both positive and negative ionization mode (respectively, ESI+ and ESI-). MS operative conditions: heat block temperature: 400°C; nebulizing gas (nitrogen) flow rate: 3 L min^-1^; drying gas (nitrogen) flow rate: 15 L min^-1^; desolvation line (DL) temperature: 250°C. Collision gas: argon (230 kPa). Transitions monitored: ESI^+^: *m/z* 355.00 →163.00 for CGAs and *m/z* 517.00 →163.00, *m/z* 517.00 →145.00, *m/z* 517.00 →135.00 for diCQAs (dwell time: 20 ms, collision energy -35 V, event time: 0.096 s); ESI^-^: *m/z* 513.00 →179.00 for CGAs and *m/z* 515.00 →179.00, *m/z* 515.00 →191.00, *m/z* 515.00 →135.00 for diCQAs (dwell time: 20 ms, collision energy: 35 V, event time: 0.096 sec). The MRM transitions were selected on the basis of the fragments obtained by analyzing the CGAs and diCQAs standards in full-scan mode in both ESI^+^ and ESI^-^ in the range of 300–1200 *m/z*, with a scan speed of 1000 μ sec^-1^ and then in product ion scan mode in both ESI+ and ESI - in the range of 100–550 *m/z*, with a scan speed of 1000 μ sec^-1^ and using as precursor ions: 355.00 *m/z* [M+H]^+^ for ESI^+^ and 353.00 *m/z* [M-H]^-^ for ESI^-^ for CGAs and 517.00 *m/z* [M+H]^+^ for ESI^+^ and 515.00 *m/z* [M-H]^-^ for ESI^-^ for diCQAs.

For the quantification of CGAs and diCQAs the external calibration method based on the following transitions: ESI+: *m/z* 355.00 →163.00 for CGAs and *m/z* 517.00 →163.00 for diCQAs was adopted. A five points calibration curve was built for CGAs analyzing in triplicate the pure standards in the range of 5–500 μg mL^-1^ while a four points calibration curve was built for diCQAs analyzing in triplicate the pure standards in the range of 5–100 ng mL^-1^. The determination coefficient (R^2^) was in all cases higher than 0.992.

Chlorogenic acid (CGA), neochlorogenic acid (neoCGA), cryptochlorogenic acid (cryptoCGA), and CoA were purchased from Sigma–Aldrich, while the necessary diCQAs (1,3; 1,5; 3,5; 3,4; 4,5 isomers) were provided from TransMIT (Marburg, Germany).

VIGS silenced artichoke grinded tissues (100 mg) was suspended in 300 μl of 70% (v/v) methanol and sonicated for 20 min in a water bath. After centrifugation (10,000 *g* for 10 min), the supernatant was filtered with a 13 mm diameter, 0.22 μm pore diameter PTFE syringe filter and analyzed on a Shimadzu XR system equipped with a photodiode detector SPD-M20A (Shimadzu, Dusseldorf Germany). An Ascentis^®^ Express C18 column (150 mm × 2.1 mm i.d., 2.7 μm particle size, Supelco, Bellefonte, PA) was used and the analysis conditions were: mobile phase: eluent A: 0.1% formic acid in water; eluent B: 0.1% formic acid in acetonitrile; mobile phase gradient was as follows: 5–25% B in 10 min, 25–40% B in 5 min, 40–100% B in 5 min and 100% B for 1 min. Injection volume: 5 μL; the flow rate of the mobile phase was 0.4 mL min^-1^ and the column was maintained at 30°C. UV spectra were acquired in the 210–450 nm wavelength range. The identification of the CQAs was carried out by comparing retention times and UV spectra with those of the commercially available standards. For the quantification of CGA and 3,5-diCQA the external calibration method based on the LC-PDA profiles acquired at 325 nm was adopted. A four points calibration curve was built for both compounds analyzing in triplicate the pure standards in the range of 1–100 μg/ml for CGA and in the range of 0.5–10 μg/ml for 3,5-diCQA. The determination coefficient (R^2^) were 0.998 for CGA and 0.999 for 3,5-diCQA.

Mean comparison was conducted using Tukey’s test. All the data were statistically analyzed using SPSS statistical software.

### Subcellular Localization Studies

The full length sequences of the *HQT1, HQT2, HQT3* genes were amplified from globe artichoke cDNA using attB specific primers (Supplementary Table 1) and recombined into the pDONR207 Entry vector through a Gateway strategy. The amplicons were cloned into pK7WGF2 (Karimi et al., 2002) producing pK7-35S:GFP:HQT1, pK7-35S:GFP:HQT2, and pK7-35S:GFP:HQT3, respectively. As a control, the unmodified vector for expression of *GFP*, pK7WGF2 (under the control of the 35S promoter), and an endoplasmic reticulum-targeted pBIN-GFP-KDEL construct were also agro-infiltrated into *Nicotiana benthamiana* leaves. The expression constructs pK7-35S:GFP:HQT1, pK7-35S:GFP:HQT2, and pK7-35S:GFP:HQT3, and the pK7WGF2 and pBIN-GFP-KDEL vectors alone (controls) were transformed into *Agrobacterium tumefaciens* strain C5801. The obtained recombinant *A. tumefaciens* strains were grown at 28°C and 220 rpm for 24 h in 5 mL of L media containing spectinomycin (100 mg L^-1^) and tetracycline (10 mg L^-1^). The overnight cultures (2 mL) were then transferred into 20 mL of induction medium [L broth containing 10 mM MES and 20 μM acetosyringone with spectinomycin (100 mg L^-1^)], and grown as above. The cells were collected by centrifugation at 4,000 g and resuspended in 50 mL of infiltration medium (10 mM MgCl_2_, 10 mM MES, 200 μM acetosyringone) to an OD600 of 1.0 and kept at room temperature for 3 h before being infiltrated into the abaxial air spaces of 5-week-old *N. benthamiana* plants. *N. benthamiana* plants were grown from seeds on soil in a climate chamber at 25°C (16 h light)/25°C (8 h dark). The localization of fluorescent proteins was analyzed 4 days post-agroinfiltration in small leaf samples (1 cm^2^ leaf explant from at least three independent agro-infiltrated plants) by confocal laser scanning microscopy. All images were acquired and processed using a Leica TCS SP2 confocal microscope and software (Leica Microsystems GmbH, Wetzlar, Germany) as described in Eljounaidi et al. (2015). GFP and plastid fluorescence were both excited at 488 nm with emission recorded at 500–525 nm and 600–640 nm, respectively. A scanning resolution of 1024 × 1024 pixels was chosen and serial optical sections were acquired with either 1 or 2 μm resolution along the *z*-axis. Quantification of transgene expression in transformed *N. benthamiana* plants was performed by a semi-qPCR analysis (as described in Transient Heterologous Expression in *N. benthamiana*).

## Results

### Genome-Wide Identification and Phylogenetic Analysis of Globe Artichoke BAHD Acyltransferases

**FIGURE 2:**
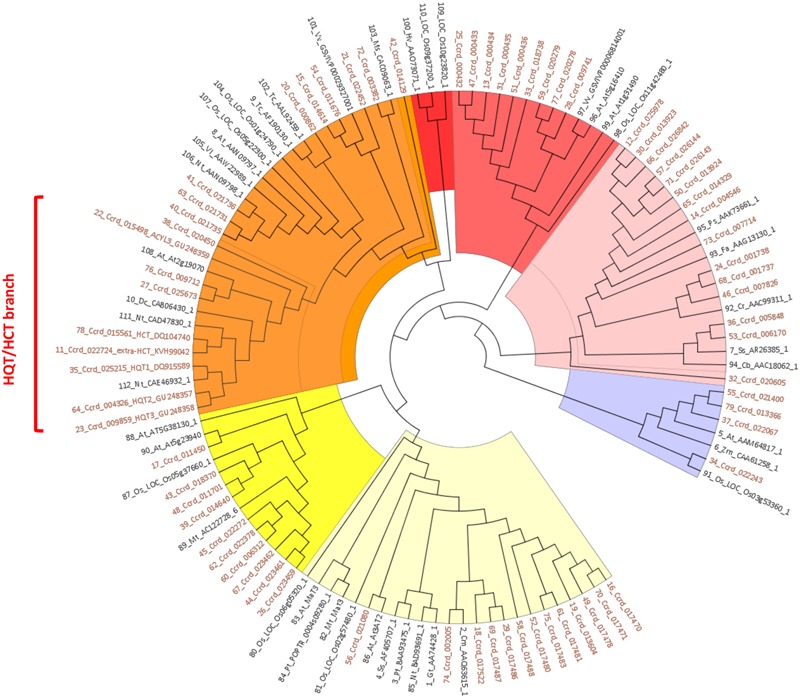
**UPGMA based phylogenetic tree was constructed of 69 globe artichoke BAHD members sequences and 43 fully/partially characterized sequence from other species.** The colors for highlighting clades are: violet (Clade I), pink (Clade II), light red (Clade III), red (Clade IV), orange (Clade V), yellow (Clade VI), and pale yellow (Clade VII). Sequences used for building the tree are reported in the Data Sheet 1.

A survey of the globe artichoke genome (release v1.0, Scaglione et al., 2016), showed the existence of 74 genes with high similarity to previously characterized BAHD acyltransferases (Menin et al., 2010; Tuominen et al., 2011). By considering only those carrying the catalytic site HXXXD, the number was reduced to 69. A phylogenetic analysis of these 69 globe artichoke BAHD proteins together with 43 representative proteins of the BAHD family from 21 species highlighted seven major clades (**Figure 2**). Four globe artichoke proteins clustered into Clade I, corresponding to the group classified as Clade II by Tuominen et al. (2011), defined by the characterized Glossy2 and CER2 homologs in *Zea mays* (Tacke et al., 1995) and *A. thaliana* (Negruk et al., 1996; Xia et al., 1996), involved in the extension of long chain epicuticular waxes, which are important both for restricting water loss and for defence against pathogens. Clade II contained 15 globe artichoke proteins and is sister to the Clade IIIa reported by Tuominen et al. (2011), along with acyltransferases which utilize a range of alcohol substrates to produce volatile esters (D’Auria, 2006). Nine globe artichoke proteins were grouped in Clade III, corresponding to the Clade IIIb described in Tuominen et al. (2011), which lacked any functionally defined homolog. No globe artichoke BAHD proteins occurred in Clade IV which contained sequences related to barley agmatine coumaroyl transferase (ACT), an enzyme involved in the biosynthesis of anti-fungal hydroxycinnamoyl agmatine derivatives (Burhenne et al., 2003). Clade V can be subdivided further into several subgroups, as reported in D’Auria (2006), three of which contained characterized enzymes: the first clustered three globe artichoke proteins along with enzymes that are involved in biosynthesis of volatile esters; the second grouped one globe artichoke protein with enzymes, from *Taxus* species, involved in the production of the compound paclitaxel; the third clustered nine globe artichoke sequences with enzymes that use hydroxycinnamoyl/benzoyl CoA as acyl donor. Clade VI is sister to the group classified as Clade Ib by Tuominen et al. (2011) and includes ten globe artichoke members which lack any functionally defined homolog. Finally, Clade VII, corresponding to the group classified as Clade Ib by Tuominen et al. (2011), grouped 13 globe artichoke paralogous proteins along with the characterized enzymes involved in modification of phenolic glycosides, predominantly anthocyanins (Suzuki et al., 2002).

We focused our attention on Clade V, in particular on the sub-group containing the biochemically characterized hydroxycinnamoyltransferases (HCT/HQT) involved in biosynthesis of lignin and chlorogenic acid. In this sub-group the globe artichoke sequences included are: HCT (AAZ80046, Comino et al., 2007), Acyltransf_1 and Acyltransf_2 (ADL62854, ADL62855.1, Menin et al., 2010; renamed in this paper as HQT2 and HQT3, respectively) and HQT (ABK79689, Comino et al., 2009; renamed in this paper as HQT1) plus an extra-HCT (KVH99042) functionally predicted *in silico*. The remaining four predicted sequences are related to previously characterized enzymes: KVI01309.1 (locus *Ccrd_020450*) and KVI06149.1 (locus *Ccrd_015498*) appeared similar to an ω-hydroxypalmitate O-feruloyl transferase (HHT1, Lotfy et al., 1995, 1996), while KVI11866.1 (locus *Ccrd_009712*) and KVH57169.1 (locus *Ccrd_025673*) are similar to a spermidine hydroxycinnamoyl transferase (SHT, Grienenberger et al., 2009).

**FIGURE 3:**
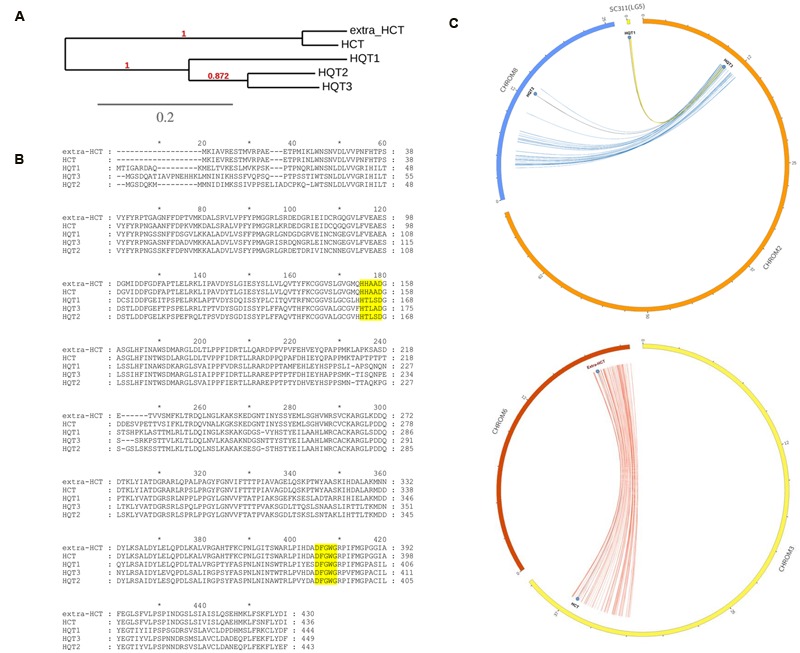
**Phylogenetic and bioinformatic analyses on BAHD. (A)** UPGMA based phylogenetic tree of five globe artichoke BAHD involved in biosynthesis of lignin and chlorogenic acid and belonging to HQT/HCT branch of the Clade V. **(B)** Muscle alignment of the CQAs related proteins (in yellow the two functional domains). **(C)** CQAs related ohnologous genes distribution over the globe artichoke pseudomolecules.

Many BAHD multiple gene copies were observed in the globe artichoke genome, and the five CQA-related genes appeared pairwise highly similar (**Figure 3A**), exhibiting the functional HXXXD or DFGWG domains (**Figure 3B**); they appeared as duplicated genes, but resident in different chromosomes in syntenic segments, likely fruit of a WGD event. In particular, *HQT2* (in Chr8), *HQT3* (in Chr2), and *HQT1* (unplaced scaffold 311, formerly mapped on Lg5/Chr5, Comino et al., 2009) appeared as ohnologous genes (Ohno et al., 1968), in the same way as for the *HCT* (Chr3) and extra-*HCT* (Chr6) genes, as depicted in **Figure 3C**. Only HQT-like enzymes involved in CGA synthesis were selected for *in vivo* functional investigation.

### Expression of HQT-like Acyltransferases in Different Globe Artichoke Tissues

**FIGURE 4:**
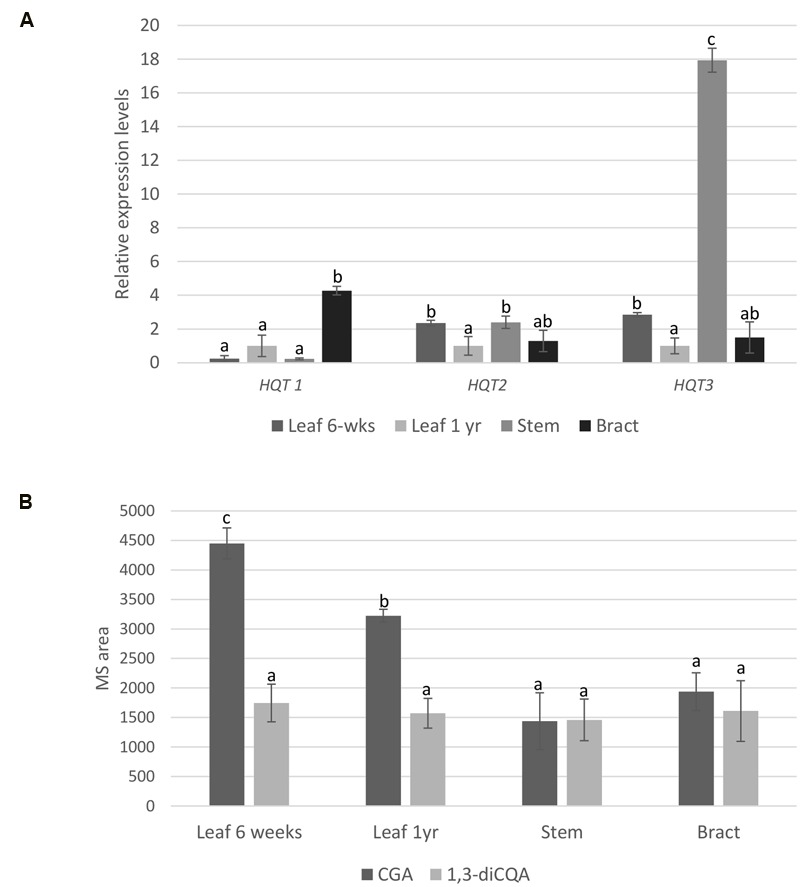
**qPCR and LC-MS analyses. (A)** Relative gene expression of *HQT1, HQT2*, and *HQT3* in globe artichoke leaf (6-weeks, 1 year), stem and bract tissues. Globe artichoke actin was used as reference gene. Error bars represent SD (*n* = 3). Different letters associated with the set of means indicate significance based on Tukey’s test (*P* ≤ 0.05). **(B)** Relative concentration of chlorogenic acid (CGA) and 1,3-dicaffeoylquinic acid (1,3-diCQA) in globe artichoke (F1 hybrid ‘Concerto’) tissues (leaf from 6 weeks-old plants and from 1 year-old plants, stem, bract). Concentrations were compared by measuring mass signals of the molecular ion ([M+H]) in different tissues. Different letters associated with the set of means indicate significance based on Tukey’s’s test (*P* < 0.05).

Expression of the genes encoding for HQT1, HQT2, and HQT3 was analyzed by qPCR and compared to the levels of CQAs at the same stage of plant development. The gene expression profiles are shown in **Figure 4A**. The expression profile for *HQT1* was higher in bract, with a level of expression 4.3-fold higher than 1-year leaf tissue. Expression levels of *HQT3* were notably high in stem tissue, where transcripts were 17-fold more abundant than 1-year leaf tissue. Expression of *HQT2* was greatest in both stem and 6-week leaf tissues.

To evaluate any relationship between gene expression and metabolite content, CGA and 1,3-diCQA were quantified in the same globe artichoke tissues by liquid chromatography coupled to mass spectrometry (LC-MS) of methanol extracts of freeze dried plant material, and compared to two original standards. Significant differences in CGA contents were observed (*p* ≤ 0.05) among leaf and other tissues (**Figure 4B**), but statistical differences in 1,3-diCQA contents were not found for any of the analyzed tissues.

### VIGS in Globe Artichoke

Four weeks after infiltration, plants inoculated with pTRV1 and pTRV2 vector showed no obvious differences compared with the control in overall shoot and leaf morphology. The virus was detected in plants agro-infiltrated with pTRV2 vector, while no virus was detected by PCR in control plants (data not shown).

**FIGURE 5:**
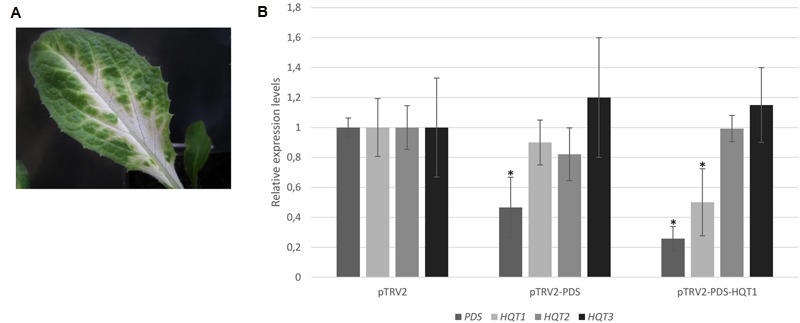
**VIGS of globe artichoke genes using pTRV vectors.** Globe artichoke seedlings were infected with *Agrobacterium* transformed with pTRV2-PDS vectors. Photographs of the leaves were taken 5 weeks after infiltration **(A)**. Relative transcript levels of *PDS, HQT1, HQT2, HQT3* genes were evaluated through qPCR analysis **(B)** in pTRV2, pTRV2-PDS, and pTRV2-PDS-HQT1 infected leaves. ^∗^*P* ≤ 0.05 vs pTRV2 infected (control).

To determine if endogenous gene silencing can also be elicited by TRV-mediated VIGS, we inserted PDS marker into a pTRV2 VIGS vector. 4 weeks after infiltration, a photobleached phenotype was observed, mainly localized in proximity of the main veins of young leaves of ‘Concerto’ globe artichoke seedlings (**Figure 5A**). No photobleaching phenotype was observed in plants infected with pTRV2 empty vector. To monitor the silencing level of *PDS*, a qPCR analysis was performed. The results revealed that *PDS* transcript levels in photobleached leaves were reduced by more than 50% compared to the controls (**Figure 5B**). The VIGS approach was applied for silencing the *HQT*-like acyltransferases involved in the final steps of the caffeoylquinic acid pathway. To achieve single gene silencing (i.e., to avoid post-transcriptional silencing of closely related gene sequences), sequence identity of more than 22 nt with other genes has to be avoided (Gaquerel et al., 2013). This pre-requisite was achieved for *HQT1*, while due to the high level of identity between *HQT2* and *HQT3* it was not possible to perform single gene silencing on these genes. ‘Concerto’ globe artichoke seedlings were infiltrated with a mixture of *Agrobacteria* transformed with pTRV2-PDS-HQT1. The photobleached leaf phenotype correlated with a marked reduction in the expression level of the genes introduced into the silencing vector. *PDS* transcript levels in photobleached leaves were 70% compared to the control. The *HQT1* transcript levels in leaves were reduced to 50% of those found in the control (**Figure 5B**). No cross-silencing of *HQT2* and *HQT3* was detected in *HQT1* silenced leaves (**Figure 5B**).

**FIGURE 6:**
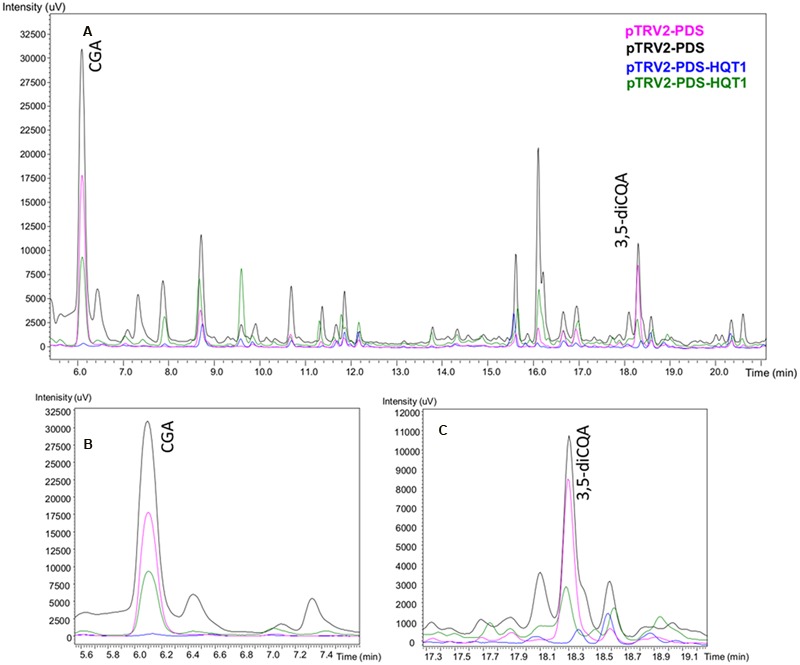
**LC-PDA profiles of leaf extracts of globe artichoke agro-infiltrated with *Agrobacterium* transformed with different TRV vectors.** Pink/black (pTRV2-PDS biological replicates), Blue/Green (pTRV2-PDS-HQT1 biological replicates) **(A)**. The major peaks correspond to chlorogenic acid **(B)** and 3,5-dicaffeoylquinic acid **(C)**.

The VIGS-silenced leaves were analyzed by (LC)-PDA, comparing their UV profiles to those obtained from a CQA standards mixture. The silencing of *HQT1* (**Figure 6**) resulted in a significant reduction in content of both chlorogenic and 3,5-dicaffeoylquinic acids (33,6 ± 31,3 vs 184,85 ± 59,65 μg/g FW and 2,35 ± 0,95 vs 18,8 ± 2,1 ng/g FW, respectively).

### *In vivo* Expression of HQT-Like Acyltransferases in *N. benthamiana*

*Agrobacteria* transformed with a pEAQ expression vector containing *HQT1, HQT2*, and *HQT3* were infiltrated into *N. benthamiana* leaves. Plants agro-infiltrated only with the empty vector were used as negative controls. 4 days after infiltration, transformed leaves were assayed for monoCQA and diCQAs content by LC-MS analysis.

**FIGURE 7:**
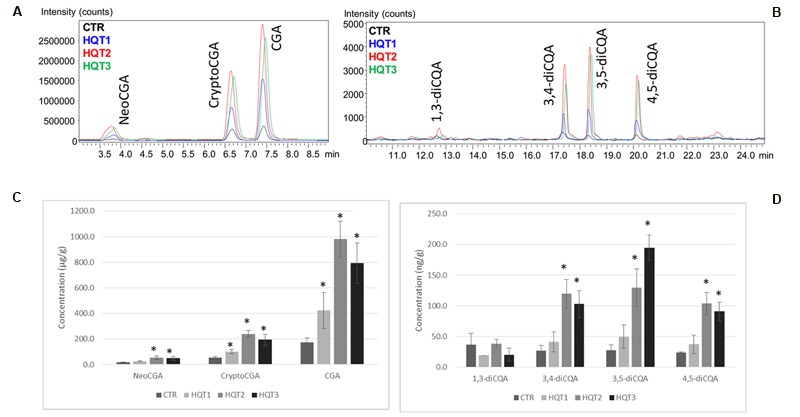
**LC-MS ESI^+^ MRM profiles. (A)** monoCQAs (NeoCGA, CryptoCGA, CGA) and **(B)** diCQAs (1,3-diCQA, 3,4-diCQA, 3,5-diCQA, 4,5-diCQA) in *Nicotiana benthamiana* control (CTR) and transformed tissues extracts (HQT1, HQT2, and HQT3). Concentration of **(C)** monoCQAs (NeoCGA, CryptoCGA, CGA) and **(D)** diCQAs (1,3-diCQA, 3,4-diCQA, 3,5-diCQA, 4,5-diCQA) in tissue extracts of *N. benthamiana* control (CTR) and transiently transformed leaves (HQT1, HQT2, and HQT3). Error bars represent SD (*n* = 3). Asterisk indicates significance based on Tukey’s test (*P* ≤ 0.05).

*N. benthamiana* leaves transiently expressing HQT1, HQT2, and HQT3 were characterized by a significant increase in mono and diCQAs content (**Figure 7**, Supplementary Image 1). In particular the overexpression of HQT1, HQT2, and HQT3 determined a 2–5 fold increase on mono CQAs and a 1–4 fold increase for diCQAs. The diCQA content in tissue extracts of transiently transformed leaves is between 6.4 and 64.9 ng/mL while the mono CQAs content is 2,000–4,000 higher ranging between 8.7 and 326.8 μg/mL (**Figure 7**). Transient transformants and controls (plants transformed with empty vector) were also tested for expression of the *HQT1, HQT2*, and *HQT3* transgenes by semi-qPCR using specific primers for globe artichoke *HQT1, HQT2*, and *HQT3* sequences. Expression of the transgene was demonstrated in all *HQT1, HQT2*, and *HQT3* transformants, while no amplification was detected in control pEAQ-HT transformed plants (Supplementary Image 2).

### Subcellular Localization Studies of HQT1, HQT2, and HQT3

No putative targeting sequences predicting mitochondrial, plastid and ER localization were found for HQT1, HQT2, and HQT3 proteins. For the *in vivo* assessment of subcellular localization of the enzymes, *N. benthamiana* plants were infiltrated with *Agrobacteria* suspension harboring the expression constructs pK7-35S:GFP:HQT1, pK7-35S:GFP:HQT2, pK7-35S:GFP:HQT3 as well as the pK7WGF2 and the endoplasmic reticulum-targeted pBIN-GFP-KDEL vector as controls. Expression of the fusion genes was confirmed using semi qPCR with gene specific primers (Supplementary Image 3).

**FIGURE 8:**
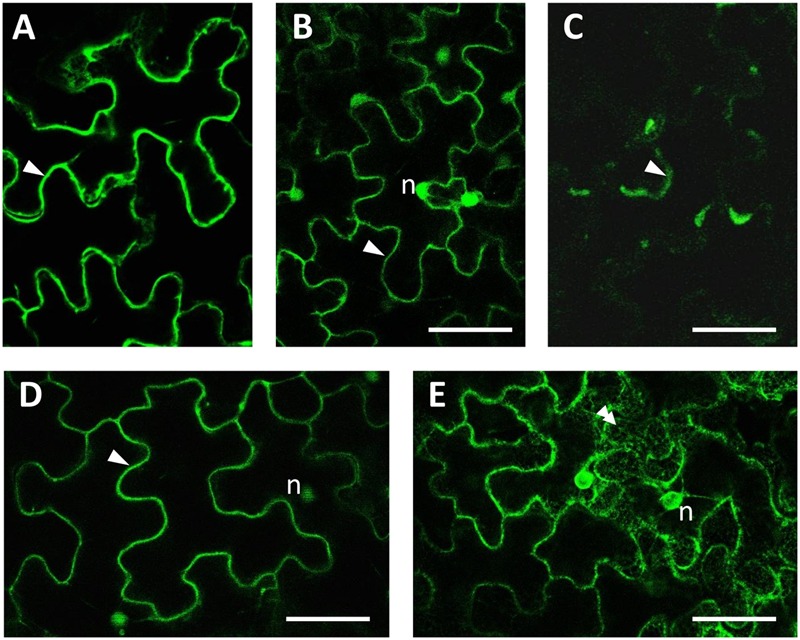
**Subcellular imaging of GFP-tagged constructs expressed in *N. benthamiana* agroinfiltrated leaves.** Confocal microscopy observations of leaf epidermal cells showed that the fluorescence pattern for GFP:HQT1 **(A)**, GFP:HQT2 **(B)** and GFP:HQT3 **(C)** was compatible with their localization in the cytosol (arrowheads). For reference, the localization of free GFP in both the cytoplasm and nucleus, and the lace-like fluorescence pattern of ER-localized GFP-KDEL are presented in **(D,E)**, respectively. (n) = nucleus; bars = 50 μm.

The subcellular localization of each protein was analyzed by confocal laser scanning microscopy (**Figure 8**). All three GFP-tagged proteins accumulated at the periphery of the cells and in cytoplasmic strands (**Figures 8A–C**). Significant differences in protein distribution can anyway be highlighted: GFP:HQT1 (**Figure 8A**) appeared to be excluded from the nucleoplasm (**Figure 8A**); GFP:HQT2 (**Figure 8B**), by contrast, also diffused in the nucleus, generating a very similar pattern to that observed for free cytosolic GFP (**Figure 8D**) but different from the localization of GFP-KDEL in the endoplasmic reticulum (**Figure 8E**); lastly, GFP:HQT3 localization (**Figure 8C**) can be described as cytosolic, even if the transient expression of this construct resulted in a weaker accumulation of fluorescent signal compared to the previous two fusion proteins.

Confocal microscopy imaging of GFP fusion constructs is compatible with the presence of the chimeric proteins in the cytoplasm rather than in other organelles, such as the vacuole or the endoplasmic reticulum, in line with with the *in silico* predicted localization of HQT1, HQT2, and HQT3 proteins in the cytoplasm.

## Discussion

Globe artichoke is a rich source of compounds important for their pharmaceutical and nutritional properties. The beneficial effects of globe artichoke for human health are mainly due to its content of flavonoids and phenolic acids, particularly caffeic acid and its derivatives mono- (e.g., chlorogenic acid, CGA) and diCQAs. These compounds make the species very attractive as a source of health-promoting molecules, both by direct consumption of the edible part, fresh or cooked, and also for industrial scale extraction of antioxidants and food additives.

Two classes of hydroxycinnamoyltransferases, the hydroxycinnamoyl-CoAshikimate/quinate hydroxycinnamoyl transferases (HCT) and the hydroxycinnamoyl-CoA:quinate hydroxycinnamoyl transferases (HQT) have been demonstrated *in vitro* to synthesize CGA in globe artichoke (Comino et al., 2007, 2009; Menin et al., 2010). These enzymes belong to the BAHD hydroxycinnamoyltransferase family (the name derives from the initial letters of the first four enzymes characterized from this family, St-Pierre and De Luca, 2000) and they transfer an acyl group from a Coenzyme-A activated hydroxycinnamic acid (e.g., cinnamoyl-CoA, 4-*p-*coumaroyl-CoA, or caffeoyl-CoA) to an acceptor molecule.

Phylogenetic analyses have been performed on BAHD sequences previously: D’Auria (2006) identified five major phylogenetic clades based on 46 biochemically or genetically characterized members; while Tuominen et al. (2011) identified eight clades based on 69 biochemically characterized plant BAHD acyltransferases and putative members from *Populus, Arabidopsis, Oryza*, and *Medicago* plants. Recently, a reference globe artichoke genome sequence has been released (Scaglione et al., 2016), allowing us to perform genome-wide analysis with the goal of identifying all sequences containing the BAHD acyltransferase conserved catalytic domain HXXXD, and the key functional domain DFGWG (D’Auria, 2006). In a previous study, 32 globe artichoke BAHD unigenes were found to cluster in 6 main clades (Menin et al., 2010), while in our study 69 putative BAHD sequences were found to cluster in seven main clades named accordingly to the nomenclature proposed by Tuominen et al. (2011).

A subgroup of Clade V contained nine BAHD genes, of which five (**Figure 3B**) belong to one or other of the two main classes of HQT/HCT-like proteins, forming two specific ohnologous groups. These are the HCT group: Ccrd_022724 and Ccrd_015561; and the HQT group: Ccrd_025215 (HQT1), Ccrd_004326 (HQT2), and Ccrd_009859 (HQT3). The three globe artichoke HQTs (here named HQT1, HQT2, and HQT3) correspond to those isolated earlier: HQT (Comino et al., 2009), and Acyltransf_1 and Acyltransf_2 (Menin et al., 2010). Previous *in vitro* characterizations highlighted their involvement in CQA biosynthesis, due to their ability to use either *p*-coumaroyl-CoA or caffeoyl-CoA as an acyl donor and quinic acid as an acceptor. The presence of 3 *HQT*-like and two *HCT*-like genes is presumably the result of a duplication event at the chromosomal level (**Figure 3C**), which likely occurred during plant evolution. As previously reported (Scaglione et al., 2016), the Asteraceae family and thus subsequent lineages including globe artichoke (Barker et al., 2008) experienced one WGD at approximately 40–45 My. WGD events, driving gene family extension and promoting functional diversification, encouraged novelty and success in many plants that are now crops, mainly in regard to metabolic pathways including glucosinolates, methyltransferases, fruit-controlling genes, and resistance gene analogs (Lei et al., 2012; Sato et al., 2012; Hofberger et al., 2013; Kim et al., 2014). In globe artichoke (Scaglione et al., 2016) the occurrence of the *HCT/HQT* duplication might have favored a high accumulation of chlorogenic acid and diCQAs through the diversification of appropriate biosynthetic functions. Indeed the presence of several homologous genes has already been described in plants belonging to the Asteraceae family, such as sunflower, lettuce and more recently in chicory, where two *HCTs* and three *HQTs* have been characterized (Legrand et al., 2016). Detection of the same number of *HCT/HQT* genes in both globe artichoke and chicory seems fully in accordance with the wide conserved syntenic regions recently observed within the family (Scaglione et al., 2014).

Hydroxycinnamoyl-CoA:shikimate/quinate hydroxycinn-amoyl transferases have been demonstrated *in vitro* to acylate a wide variety of acceptors, including shikimate, quinate (Hoffmann et al., 2003), 3′-hydroxyanthranilate (Moglia et al., 2010), gentisate, 2,3-dihydroxybenzoate, catechol, protocatechuate, 5-hydroxyanthranilate, 3-hydroxybenzoate, 3-aminobenzoate and hydroquinone (Eudes et al., 2016) and seem more related to the lignin pathway, as *in vivo* experiments have shown their key role in the synthesis of the lignin monomers, coniferyl and sinapyl alcohols. Indeed, downregulation of *HCT* in *N. benthamiana, Arabidopsis thaliana* (Hoffmann et al., 2004) and in *Pinus radiata* (Wagner et al., 2007) altered their lignin composition and content while a natural *HCT* mutant of poplar (*Populus nigra*) also showed an altered lignin composition (Vanholme et al., 2013).

Hydroxycinnamoyl-CoA:quinate hydroxycinnamoyl transferases have been shown to be directly involved in CGA biosynthesis in tobacco, tomato, and globe artichoke (Niggeweg et al., 2004; Comino et al., 2009; Menin et al., 2010); their down-regulation in *N. benthamiana* and in both tomato (Niggeweg et al., 2004) and potato (Payyavula et al., 2015) leads to a considerable reduction in CGA content.

Since our main objective was to evaluate the *in vivo* physiological roles of CGA biosynthetic genes in globe artichoke, we focused our analysis on three HQT-like enzymes, HQT1, HQT2, and HQT3. When their transcription levels were analyzed in different globe artichoke tissues, both HQT2 and HQT3 were found highly expressed in vegetative leaves and stem, while HQT1 was located in bract tissues (**Figure 4A**). The CQA content in plant is influenced not only by BAHD acyltransferases but also from structural genes of phenylpropanoid pathway (PAL, 4CL, C4H) and regulatory genes (such as MYB transcription factors). The differential accumulation of CQAs, observed in the analyzed tissues, and the lack of a direct correlation with HQTs suggests that the additional gene(s) required for the biosynthesis of these compounds are probably involved.

Although micropropagation and *in vitro* callogenesis techniques (Menin et al., 2013) have been applied in globe artichoke, efficient protocols for the establishment of *in vitro* organogenesis, a pre-requisite for *Agrobacterium*–mediated genetic transformation, have not yet been developed. For this reason, a rapid system for transient transformation is highly desirable to expedite gene function analysis in globe artichoke. VIGS has been used routinely for analysis of gene function in many plant species (Baulcombe, 1999; Burch-Smith et al., 2004), mainly because is a robust method that avoids the need for laborious and time-consuming generation of stable transformants. The effectiveness of VIGS as a strategy to validate the physiological role of genes has been demonstrated in many species such as opium poppy (Wijekoon and Facchini, 2012), *Withania somnifera* (Singh et al., 2015), peach (Bai et al., 2016), cotton (Zhu et al., 2015), and *Litchi chinensis* (Li et al., 2016). The most widely used VIGS vectors are derived from the Tobacco Rattle Virus, which invades a wide range of hosts and spreads vigorously throughout the entire plant (Senthil-Kumar and Mysore, 2014). Within the Asteraceae family the TRV-based VIGS system was applied for the first time in Gerbera (Deng et al., 2012).

Because TRV is a “mosaic” virus, it is helpful to have an easily visible reporter/marker in order to determine whether the target gene has been silenced (Chen et al., 2004). In our experiments, we used tandem constructs containing PDS as reporter and a target gene as a tool for examining the function of chlorogenic acid-associated genes. The *PDS* gene has been widely used as a VIGS marker in various plant species as it encodes an enzyme required for the biosynthesis of carotenoids, which in turn protect chlorophyll from photo-oxidation. Silencing of *PDS* results in decreased carotene content and ultimately to the easily observed outcome of leaf photobleaching.

Four weeks after agro-infiltration, around 20% of globe artichoke plants treated with pTRV2-PDS developed typical photobleaching symptoms on the upper newly-grown leaves (**Figure 5A**). The silencing efficiency was lower than in model species, such as *N. benthamiana* and tomato, but comparable to that observed in *Papaver somniferum* (23%) (Hileman et al., 2005), *Populus tormentosa* (30%) (Jiang et al., 2014), *Gerbera hybrida* (35%) (Deng et al., 2012), and *Aquilegia* (12%) (Gould and Kramer, 2007).

The globe artichoke leaves containing down-regulated *HQT1* accumulated reduced amounts of both chlorogenic and 3,5-dicaffeoylquinic acids (**Figure 6**), thus confirming the physiological function of HQT1 enzyme previously characterized *in vitro* (Comino et al., 2009). An opposite trend was observed upon transient over-expression in *N. benthamiana*. These results clearly confirm that HQT1, HQT2, and HQT3 are involved in the synthesis of CGA. Interestingly, the increased accumulation of diCQAs observed in extracts from *N. benthamiana* over-expressed plants might be a consequence of enzymatic conversion of CGA to diCQAs. Our metabolic results are in accordance with those of Sonnante et al. (2010), who achieved both transient and stable over-expression in *Nicotiana* of HQT3 - named hqt1 in that paper.

Several members of the BAHD family have been shown to be cytosolic (Fujiwara et al., 1998; Yu et al., 2008) with some exception like as CER2 that it was shown to be ER localized (Molina and Kosma, 2015). An *in silico* analysis using Target P predicts a cytoplasm destination for HQT1, HQT2, and HQT3; these predictions were confirmed for HQT1, HQT2, and HQT3. A recent study (Moglia et al., 2014) proposed that tomato HQT localizes to vacuoles as well as to the cytoplasm of plant cells, supporting the idea that in this species the enzyme catalyzes different reactions in two separate sub-cellular compartments.

This work is a further contribution to the understanding of the genetic basis of biosynthesis of CQAs in globe artichoke through *in vivo* functional studies. Thanks to the development of VIGS in globe artichoke, described in this work, achieving functional genomics in this species will become much easier and quicker. These tools and results, together with the recently published globe artichoke reference genome sequence, will greatly facilitate the development of a new generation of globe artichoke varieties with enhanced bioactive properties that can significantly contribute to better human nutrition. Furthermore, the present results are also of interest for developing microbial and plant-based platforms for the production of these pharmaceutically relevant secondary metabolites.

## Author Contributions

AM and CCo designed and planned the experiments and co-wrote the paper. AA performed the bioinformatic analyses on the globe artichoke genome. CCa and PR undertook the LC-PDA-MS/MS analyses on transiently transformed *N. benthamiana* and on VIGS silenced globe artichoke tissues. KE, KC, and JB performed LC-QTOF-MS analysis in globe artichoke tissues and set up the VIGS protocol in globe artichoke. AMM and AM performed the cloning and agro-infiltration experimental work for VIGS and over-expression experiments. AG performed the sub-cellular localisation imaging. All the authors drafted the manuscript and approved its final version.

## Conflict of Interest Statement

The authors declare that the research was conducted in the absence of any commercial or financial relationships that could be construed as a potential conflict of interest.

## Footnotes

1 http://www.ebi.ac.uk/Tools/msa/muscle/

2 http://mafft.cbrc.jp/alignment/server

3 http://tree.bio.ed.ac.uk/software/figtree

4 https://genomevolution.org

5 http://bioinfo.ut.ee/primer3/
