# Supplementary material for: Genome-Wide Identification of BAHD Acyltransferases and In vivo Characterization of HQT-like Enzymes Involved in Caffeoylquinic Acid Synthesis in Globe Artichoke
Source: Front Plant Sci. 2016 Sep 23;7:1424. doi: 10.3389/fpls.2016.01424 (PMC5033976; doi:10.3389/fpls.2016.01424)
Supplement: Supplementary file 1 [file Table_1.DOCX]

**Supplementary Table 1**

Oligonucleotide sequences used in this work

| **HQT1_attB1** | 5’-GGGGACAAGTTTgtacaaaaaagcaggctCGATGACTATCGGAGCTCGTGA-3’ |
| --- | --- |
| **HQT1_attB2** | 5’-GGGGACCACTTTGTACAAGaaagctgggtCCTAGAAGTCATACAAGCATT-3’ |
| **HQT2_attB1** | 5’-GGGGACAAGTTTGTACAAAAAAGCAGGCTCGATGGGAAGTGATCAAAAGATGATG-3’ |
| **HQT2_attB2** | 5’-GGGGACCACTTTGTACAAGAAAGCTGGGTCTCAAAACTCGTACAAGAACTTTTC-3’ |
| **HQT3_attB1** | 5’-GGGGACAAGTTTgtacaaaaaagcaggctCGATGAACATCAATATAAAACATTCG-3’ |
| **HQT3_attB2** | 5’-GGGGACCACTTTGTACAAGaaagctgggtCTTAAAAATCATATAAGAACTTCTC-3’ |
| **HQT1-Rt-For** | 5’-TAAAATGGACGATCAGTATC-3’ |
| **HQT1-Rt-Rev** | 5’-TTATGTTCAGATTTGGACTC-3’ |
| **ACT-Rt-For** | 5’-TACTTTCTACAACGAGCTTC-3’ |
| **ACT-Rt-Rev** | 5’-ACATGATTTGAGTCATCTTC-3’ |
| **HQT2-Rt-For** | 5’-CTATGACCATGTTGAATACC-3’ |
| **HQT2-Rt-Rev** | 5’-GCTTTGAGACTATTGAGTTG-3’ |
| **HQT3-Rt-For** | 5’-ACATACGAGATCCTAGCTG-3’ |
| **HQT3-Rt-Rev** | 5’-ACGTACAATTTGGTTAGTTG-3’ |
| **PDS-Rt-For:** | 5’-GCACCTGCAGGAGAATGGA-3’ |
| **PDS-Rt-Rev** | 5’-TTGTTTGCTCTGATCTGCTG-3’ |
| **PDS-EcoF** | 5’-CGGAATTCGCAGGAGCAATCTTCTCAGTGT-3’ |
| **PDS-XhoR** | 5’- GCTCGAGGGGCAGCAAGCAACTCAC-3’ |
| **HQT1-XhoF** | 5’- CCTCGAGCCTGCACCATACGTTATCCGATGG-3’ |
| **HQT1-SmaR** | 5’- TCCCCCGGGGCGGCTAAGATCTCGTACGTGCTATG-3’ |
| **Nt-EF-For** | 5’- TGAGATGCACCACGAAGCTC-3’ |
| **Nt-EF-Rev** | 5’- CCAACATTGTCACCAGGAAGTG-3’ |
