# Supplementary material for: Genome-Wide Identification of BAHD Acyltransferases and In vivo Characterization of HQT-like Enzymes Involved in Caffeoylquinic Acid Synthesis in Globe Artichoke
Source: Front Plant Sci. 2016 Sep 23;7:1424. doi: 10.3389/fpls.2016.01424 (PMC5033976; doi:10.3389/fpls.2016.01424)
Supplement: Supplementary file 2 [file Data_Sheet_1.DOCX]

>Gt_cl_1aAA74428.1MEQIQMVKVLEKCQVTPPSDTTDVELSLPVTFFDIPWLHLNKMQSLLFYDFPYPRTHFLDTVIPNLKASLSLTLKHYVPLSGNLLMPIKSGEMPKFQYSRDEGDSITLIVAESDQDFDYLKGHQLVDSNDLHGLFYVMPRVIRTMQDYKVIPLVAVQVTVFPNRGIAVALTAHHSIADAKSFVMFINAWAYINKFGKDADLLSANLLPSFDRSIIKDLYGLEETFWNEMQDVLEMFSRFGSKPPRFNKVRATYVLSLAEIQKLKNKVLNLRGSEPTIRVTTFTMTCGYVWTCMVKSKDDVVSEESSNDENELEYFSFTADCRGLLTPPCPPNYFGNCLASCVAKATHKELVGDKGLLVAVAAIGEAIEKRLHNEKGVLADAKTWLSESNGIPSKRFLGITGSPKFDSYGVDFGWGKPAKFDITSVDYAELIYVIQSRDFEKGVEIGVSLPKIHMDAFAKIFEEGFCSLS >Cm_cl_1aAAQ63615.1MASNSIVTILEQSRISSPPGTIGERSLPLTFFDIGWVPFPPVHHVFFYRFPHSKSHFLETVVPNLKHSLSLALQHFFPFASNLYVSPNADDFGVIRKPEIRHVEGDYVALTFAECSLDFNDLTGNHPRKCENFYPLVPPLGNVVKMADCVTIPLFSVQVTYFRDSGISIGMTNHHSLGDASTRLGFLKVWTSIAKSGGDQSLLMNGSLPVLDRLIDVPKLDEYRLRHTSLETFYQPPSLVGPTKKVRATFILSRTNINQLKKRVLTQIPTLEYISSFTVTCGYIWSCIAKSLVKMGEKKGEDELEQFICTADCRSRMDPPIPSTYFGNCGAPCVTTIKNVVLSSENGFVFAAKLIGEAINKMVKNKEGILKDAERWHDAFKIPARKIGVAGTPKLNFYDIDFGWGKPQKNETISIDYNGSVAINASKESTQDFEIGLCFSNMQMEAFADIFNHGLESEI >Pf_cl_1aBAA93475.1VIETCRVGPPPDSVAEQSVPLTFFDMTWLHFHPMLQLLFYEFPCSKQHFSESIVPKLKQSLSKTLIHFFPLSCNLIYPSSPEKMPEFRYLSGDSVSFTIAESSDDFDDLVGNRPESPVRLYNFVPKLPPIVEESDRKLFQVFAVQVTLFPGRGVGIGIATHHTVSDAPSFLAFITAWSSMSKHIENEDEDEEFKSLPVFDRSVIKYPTKFDSIYWRNALKFPLQSRHPSLPTDRIRTTFVFTQSKIKKLKGWIQSRVPSLVHLSSFVAIAAYMWAGITKSFTADEDQDNEDAFFLIPVDLRPRLDPPVPENYFGNCLSYALPRMRRRELVGEKGVFLAAEVIAAEIKKRINDKRILETVEKWSPEIRKALQKSYFSVAGSSKLDLYGADFGWGKARKQEILSIDGEKYAMTLCKARDFEGGLEVCLSLPKDKMDAFAAYFSLGING >Ss_cl_1aAF405707_1MTTTTTILETCHIPPPPAANDLSIPLSFFDIKWLHYHPVRRLLFYHHPSSKSQFLHTIVPHLKQSLSLALTHYLPVAGNLLYPSNTEKFPQLRYAAGDSVPVTIAESNSDFESLTGNHTRDADQFYDLLPPIPPIEEESDWKLINIFAVQITLFPGEGICIGFSNHHCLGDARSIVGFISAWGEINGIGGYEGFLSNHSDSLSLPIFDRSFINDPNKIDAIFWKVLRNIPLKTASFPLPTNRVRSTFLLRRSDIEKLKTATKSPASSFVAAAAFVWSCMVKSGDKSDENAPELFIIPADARGRVDPPIPENYFGNCIVSSVAQVERGKLAAEDGFAVAAEAIGGEIEGKLKNRDEILRGAENWMSDIFKCFGMSVLGVSGSPKFDLLKADFGWGKARKLEVLSIDGENHSMSLCSSSDFNGGLEVGLSLPRERMAAFEEVFRASIMAASGPARRSPALVEPL >At_cl_2_AAM64817.1MEGSPVTSVRLSSVVPASVVGENKPRQLTPMDLAMKLHYVRAVYFFKGARDFTVADVKNTMFTLQSLLQSYHHVSGRIRMSDNDNDTSAAAIPYIRCNDSGIRVVEANVEEFTVEKWLELDDRSIDHRFLVYDHVLGPDLTFSPLVFLQITQFKCGGLCIGLSWAHILGDVFSASTFMKTLGQLVSGHAPTKPVYPKTPELTSHARNDGEAISIEKIDSVGEYWLLTNKCKMGRHIFNFSLNHIDSLMAKYTTRDQPFSEVDILYALIWKSLLNIRGETNTNVITICDRKKSSTCWNEDLVISVVEKNDEMVGISELAALIAGEKREENGAIKRMIEQDKGSSDFFTYGANLTFVNLDEIDMYELEINGGKPDFVNYTIHGVGDKGVVLVFPKQNFARIVSVVMPEEDLAKLKEEVTNMII >Zm_cl_2CAA61258.1MVFEQHEEEAVAPGAVHGHRLSTVVPSSVTGEVDYALADADLAFKLHYLRGVYYYRSGDGLATKVLKDPMLPWLDDHFPVAGRVRRAETEGDGAPRRPYIKCNDCGVRIVEARCDRDMAEWIRDAAPGRIRQLCYDKVLGPELFFSPLLYVQITNFKCGGLALGFSWAHLIGDIPSAATCFNKWAQILSGKKPEATVLTPPNQPLQGQSPAAPRSVKQVGPIEDLWLVPAGRDMACYSFHVSDAVLKKLHQQQNGRQDAAAGTFELVSALVWQAVAKIRGDVDTVTVVRADAAGRSGKSLANEMKVGYVESAGSSPAKTDLAELAALLAKNLVDETAAVAAFQGDVLVYGGANLTLVDMEQVDLYGLEIKGQRPVHVEYGMDGVGDEGAVLVQPDADGRGRLVTAVLPGDEIDSLRAALGSALQVA >Ss_cl_3AAR26385.1MNINQINTKLIKPITPTPQNLKNYHISFLDQHVVKKYIAVVLYYQSAPDNGRLEDSLAETLVHFYPLAGRYIKTDLTVDCSDQGAEFIEAEARGDVRVTDLIGKTDTIHLCPEQYFGLDEGVDDPLLSIQVTRFSCGGATIAVSVSHRVFDVSSLETFLSAWSSASKTGGGVAPVIPSFALASLLPNKDEKFGLDSNKCQGKEQKIAVKRLLFEKRALTRLTSERTSGVRAACAVIAKALIRLDRTTHGKSRDFVVFQPINMRGRTGVPSPKNACGNMSFGSFTRRVSAKEEVGIGELVGLIGDGVRRGIAEYTEILCPDRDGRDVIIHVRNKNIKEVFKSETFVVSFTDWSKFGFYEVDFGWGRPIWSGVGPQRPRGNQTIMMRSKEGDGIEAWVHLNEDDMDLFEQDVEIKLFLS >At_cl_5a_AAN09797.1MDHQVSLPQSTTTGLSFKVHRQQRELVTPAKPTPRELKPLSDIDDQQGLRFQIPVIFFYRPNLSSDLDPVQVIKKALADALVYYYPFAGRLRELSNRKLAVDCTGEGVLFIEAEADVALAELEEADALLPPFPFLEELLFDVEGSSDVLNTPLLLVQVTRLKCCGFIFALRFNHTMTDGAGLSLFLKSLCELACGLHAPSVPPVWNRHLLTVSASEARVTHTHREYDDQVGIDVVATGHPLVSRSFFFRAEEISAIRKLLPPDLHNTSFEALSSFLWRCRTIALNPDPNTEMRLTCIINSRSKLRNPPLEPGYYGNVFVIPAAIATARDLIEKPLEFALRLIQETKSSVTEDYVRSVTALMATRGRPMFVASGNYIISDLRHFDLGKIDFGPWGKPVYGGTAKAGIALFPGVSFYVPFKNKKGETGTVVAISLPVRAMETFVAELNGVLNVSKG >Tc_cl_5a_AF190130_1MEKTDLHVNLIEKVMVGPSPPLPKTTLQLSSIDNLPGVRGSIFNALLIYNASPSPTMISADPAKPIREALAKILVYYPPFAGRLRETENGDLEVECTGEGAMFLEAMADNELSVLGDFDDSNPSFQQLLFSLPLDTNFKDLSLLVVQVTRFTCGGFVVGVSFHHGVCDGRGAAQFLKGLAEMARGEVKLSLEPIWNRELVKLDDPKYLQFFHFEFLRAPSIVEKIVQTYFIIDFETINYIKQSVMEECKEFCSSFEVASAMTWIARTRAFQIPESEYVKILFGMDMRNSFNPPLPSGYYGNSIGTACAVDNVQDLLSGSLLRAIMIIKKSKVSLNDNFKSRAVVKPSELDVNMNHENVVAFADWSRLGFDEVDFGWGNAVSVSPVQQQSALAMQNYFLFLKPSKNKPDGIKILMFLPLSKMKSFKIEMEAMMKKYVAKV >Dc_cl_5b_CAB06430.1MSIQIKQSTMVRPAEETPNKSLWLSKIDMILRTPYSHTGAVLIYKQPDNNEDNIHPSSSMYFDANILIEALSKALVPYYPMAGRLKINGDRYEIDCNAEGALFVEAESSHVLEDFGDFRPNDELHRVMVPTCDYSKGISSFPLLMVQLTRFRCGGVSIGFAQHHHACDGMSHFEFNNSWARIAKGLLPALEPVHDRYLHLRLRNPPQIKYTHSQFEPFVPSLPNELLDGKTNKSQTLFKLSREQINTLKQKLDLSSNTTTRLSTYEVVAGHVWRSVSKARGLSDHEEIKLIMPVDGRSRINNPSLPKGYCGNVVFLAVCTATVGDLSCNPLTDTAGKVQEALKGLDDDYLRSAIDHTESKPDLPVPYMGSPEKTLYPNVLVNSWGRIPYQAMDFGWGSPTFFGISNIFYDGQCFLIPSQNGDGSMTLAINLFSSHLSLFKKYFYDF >Ccrd_022724_extra-HCT_KVH99042MKIAVRESTMVRPAEETPMIKLWNSNVDLVVPNFHTPSVYFYRPTGAGNFFDPTVMKDALSRVLVPFYPMGGRLSRDEDGRIEIDCRGQGVLFVEAESDGMIDDFGDFAPTLELRKLIPAVDYSLGIESYSLLVLQVTYFKCGGVSLGVGMQHHAADGASGLHFINAWSDMARGLDLTLPPFIDRTLLQARDPPVPVFEHVEYQPAPPMKLAPKSASDETVVSMFKLTRDQLNGLKAKSKEDGNTINYSSYEMLSGHVWRSVCKARGLKDDQDTKLYIATDGRARLQPALPAGYFGNVIFTTTPIAVAGELQSKPTWYAASKIHDALAKMNNDYLKSALDYLELQPDLKALVRGAHTFKCPNLGITSWARLPIHDADFGWGRPIFMGPGGIAFEGLSFVLPSPINDGSLSIAISLQSEHMKLFSKFLYDI >Ccrd_025978MEVEVISTESVKPSSPTPPHLKTFELSLLDQLVIXPYVPXIFYYPNHNGHNILQAQEKSLALKGSLSEILTQFYPLAGTXKDDLSIDCNDVGAYYALALVRLPLNEFLSHPDLSLTDRLLPFRPSFEASGIGGXVTNVQVNIFECXGIAIGFCISHKIVDAAALYTFLKAWTNMACGSKEVVYPNLIGARSLFPAKDLWLKEASMAMCGSWLKEGMCVTKRFVFDADAISTLKAQATRNGLQNPTRVEXVSGLIWKCAMAAFKENCGFQKPSLLTHMVNLRRKLSSTLSKDLIGNLLWFTGIEAWVNMDEEEMKILQSNSELLAFASLDPSPLPKDEVVALNKPHAMNTEIDPIK >Ccrd_000434MGISGKGFSVEVIENVVVGAEEPWNDHWLPFTNLDLLVPPFNVGSFFCYNQPSHGSFATMLSTLKASLSRALTLYYPLAGEIVWNAAAGENQIHCNNQGVDFIYAVADVQLKELNFYNPDECIEGKLIPNKPRGVLSIQVTELKCGGMVIGCMFDHRAADGYSANMFISSWGDLARSETPSMLPSFRRSILNPRSPTTYSSSIDNVFAIFEPPSKPDNDQNHDGLLINRVYYIEGGQLNKIQLLANENGLRRSKLEAFTSFLWKIVALSMEDSGYRNQMCNVAIAVDGRRRVSEGDGEEKEKLMVSHFGNVLSMSCGAKRSQELSNMSLSNLATEVHEFLQTATGKDHFLDLIDWVEERRSQPLVARAFANNEMSVMVSSGQRFQIMDKMDFGWGKVAFGSCHVPSERKDSYVMTLPSPTNDKDWVVYMHMPMEHMNYIETHASHVFNPLNADYLKI >Ccrd_004546MEIKIRSIQSIKPSKPTPENLRNFRLSLLDQLASSYINLIFYYKASGEINISDRCTQLVKSLSEVLTLFYPLAGRITEDGLIVDCSDQGIKYLETQVSTRLDDFLEQGPKIDLVNQLIGAPDQVTTTLVIQVNVFDCGALVIGVSAAHKVTDTSNLVRFINEWASMNRTGESSGAFCPCIDNMASLFPAREISSSKYSLIPNDPEAIIVTKRFVFNGYTISKLRAKASSPNRKHSRVTLVASLIWKALISIDHVKSGSFRDCLLAPAINLRGKANSAISESSFGNVWTPYPIRFLQNKMEPEFVDLVNLIEDTTRNFITWLPKASSEEICTQAIACYAEAVEEVKQDKFAIFTSWCRFPIYEADFGWGKPYWASGTGSSIEIVTLMDDKHGDGIEAWVSLNEKDMYLLEQDEDLLAFTS >Ccrd_014614MVADIQIDDPTPSHLLKLSAIDSQLFLRFTIEYLLIYRPPRHHGPADRSAVTARVKDALARALVPFYPLAGRVRARSDGSSLEVVCRGQGAVFVESTADHTVSDFEVAPRYVTEWRKFLALEVTDVLKGAPPLVVQLTWLSDGSAALGIGFSHCICDGIGSVQFLNYFSDLATSCRYGSVVELKPRPKPIWERHLLDPTPYRLQTSQHPEFNRVPDLCKFSTRFNPDQLTPTSTTFHEWRVNELKSVAASTSQLSKSSLTTFEVLSAHIWRSWARALSFPPQQSLKLLFSINVRNRVKPSLPSEYYGNAIVLGCAQTTVKELTENGLGYATELIKDAKNRVDDGFVREVVESVSLNRASLVPDSVGVLILSEWSKVGLESVDFGLGRAVQVSPVCTDKYCILLPVPDDIRSVKSMVAVPSVAVGKYEYLIRAVP >Ccrd_017470MASLPILTILEQSHVSPPPATVADASLPLAFFDILWLPFSPVHHIFFYELPAVSKTHFTETIIPNLKQSLSITLQHFFPFSGNLIVFSTPTLKPEIRYVNGDSVAFTVAESTLDFNDLTGNHPRDCGKFYHLIPLLPEATKESDHVKIPVFSIQVTFFPNSGISVGMTNHHSLGDASTRFCFLKAWTSIARTGSDESFLANGTLPFFGRVVNYPKIDELYLKRVEVETTFNKDYQPPRLSGPTDKVRATFILTRTVINGLKKWVSTQLPTLPYVSSLTVLCAYTWSCVAKSRNDELEIFSIAVDCRARIDPPIPAAYFGNCVVLCLSIAKTDVLTGNEGFLNAAKLLGENLHKMLTDKTGIVKDKWPFDSLLSQGIPTTIMGVTGTTKLKFYDMDYGWGKPTKYETISIDYNDFISLSTCKESNEDLELGVCLSATEMEAFVPIFSKGLEAYL >Ccrd_011450MGCAFNHAILDGTSTWHFMSSWAQICAGSKSISVQPFLDRTQARNTRVKLDLTPPKQQNGDVATDAKPPAEAPALREKIFRFSESAIDKIKAKVNANPPEGSTKPFSTFQSLSTHIWHAATRARQLKPEDYTVFTVFADCRKRVDPPMPDSYFGNLIQAIFTVTAAGLLQANPPEFAASMIQKAIEAHDAKAIEARNKEWESNPIIFQFKDAGVNCVAVGSSPRFKVYDVDFGFGKPESVRSGANNRFDGMVYLYQGKNGGRSIDVEISLEATAMENLEKDNEFLIEE >Ccrd_017522MLTILENCRISPPPATVGERSLPLTFFDTIWLIFSPIHQVFFYEFPHSKQHFIQTIVPKIKHSLSITLQHFFPFASNLIVFPKPNHSTVARNPEIRHLEGDSVAVTFAECDLDFNDLTGNHPRNCNKFYPLVPLLPPSTKVSDYVSIPLFSVQVTLFENSGVSIGLTNHHTLCDASTRFNFLKAWTSIARNGSDELFLASGSLPFYDRVIKYPTTLDEIHLNQPGIETIKEGYQPPQLDSHTDRVRATVVLTQAHINRLKKWVRIQQPTLEYVSSFSVACAFVWSCTAKSLAHIGDKKGDDDIEMFVCAVDWRSRFDPPIPQTYFGNCVGPCITPTTKSTLLAGEKGFLVAAELFGKALSETIKNKDGMLKEAETWLKRVSAPVPSLSVSGTPKIKIYDVDFGWGKPRKHETISLDYNRSISVNACKESTTDIEIGLSFPAKQMDAFITVFERELETTCSEQE >Ccrd_013604MASLPILTVLEQSDVSPPPATVDDRSLPLTFFDISWLPFHPVHHLFFYDLPITKTHFIETVIPNLKRSLSITLQHFFPFAGNLIVFPTPTRKPEIRYLEGDSVALTVADCTLEFNDLTGNHPRDCDKFYHLIPQLPKAAKGPDHVTIPVFAVQVTLFPDCGISIGMTNHHFLADASTRFYFLKAWTSFARCGSDESFLANGTLPFFDRVVNYPKLDESYLKNAKVETFCEGYQPQSLSGPTDKVRVTLILTRTVIDRLKKWVSTQLPTLAYVSSFTVACGYFWSCIAKSRNDELQLFGFTINCRARMDPPLPAAYFGNCVVPCIAMAKTALLMNGKEGFLTAAKLLGENLHKILTDKDGVVKDIGPLDELLSQGMPTTIIGVAGTPRLHFYDLDFGWGKPRKHETISIDYNDSISLSACKESTEDLEIGVSLSATEMEAFVNIFHGGLEAYL >Ccrd_000862MDVKISQPTTVYPSQQPFTDDHILPLSHLDTDRNMNVPFRYVRAYAAANHHHPHPFDVITVALSTALVKYYPYTGSLHRRKFDGRFELHCKVGGGVTVIPATVDSPLSSVSYLDDADEEFIELLVPNPDQQTRLTHPLMLQVTRFSCGGYTLGASVHHVLCDGLGATLFFNAMAELARGAGEVTVEPVWDRSKLLGPREPARIVFPIEEVLCLDKDFVPYSELDEKVVRECFHVKDEWLDRFKILLQERSGLSFTTFEALGAFLWQARVKASKFPREEKVKFAYAINIRKLVKPPLPAGYWGNGCVPMYVQLTAGELTERPIWETAEMIKKSKRNATNEYVHSFIDFQELNYEKGINAGKRVSAFTDWRHLGHSTVDFGWGGPVTVIPLSRNLLGSVEPCFFLPYSEASQGKKDGFKVLLYLQANAVIGFRGEMEKFGSMEYA >Ccrd_022452MQRSSNEELPDCCYQENQPSLIKPSSPTPNHMLHLSNLDDQKFLRFSIKYIYLFQNSVTIHLLKLSLSRVLVDYYPLAGRLKTLLSPHDDDDHGDDQKLQVDCNGEGATNVHVKEGRKLQVGGGKRCAMVDEVEGRGKGKENGWEMTRDGGDPQRWDCVCLEVVTKLRCGGMIVCTSINHCLCDGIGTSQFLHDWAYLTTKPIDSIPITPFHSRHMFKPRSPSSHLPLLHPAFTKNVPNSTAEDSFSVNRYLHSQPLVPASLTYTASNIMRLKSQCVPSIKCTTFEVLASHTWLSWVKSLNLAPSLEVKLLFSMNIRNRVNPKIPKGYYANGFVLACAKTTVKGLVNSNLHNVVKLVQEAKLALTDDCVNSILEMLEDKNIKTDLTASLVISQWSKLGLEDLDFGEGKPLHMGPLTSDIYCLFLPVIGHPNDIRVLVSLPKGLVSKFEYYMNRFLDSNNVEATGNCEK >Ccrd_015498_ACYL3_GU248359MVTTGTYSDKSGVELIVTRMGEPTLVQPFEETEKGLYFLSNLDQNIAVIVRTIYCFKSEEKGNEMAAEVIKDALSKVLVHYHPAAGRLTISSEGKLIVDCTNEGAVFVEAEANCNIEDIGDHTKPDPMTLGKLVYDVPGAKNILEIPPLVVQVTKFKCGGFVLGLGMNHNLFDGIAAMEFISSWSRTARGLPLEVPPFLDRTILNARNPPLVEFPHDEFAEIEDVSNTVDLYKEELAYRSFCFSPDDIQRLKIKATADGDMPTCTSFEALSAFVWKARTEALQMKPDQKTKLLFAVDGRSRFEPPLPEGYSGNGIVLTNSICKAGEQIENPLSFTVKLVHEAVKMITDGYMRSAIDYFEVTRARPSLASTLLITTWSKLSFHAQDFGWGEPIMSGPVALPEKEVILFLSHGKQRKSVNVLLGLPVSAMKTFEELMKHI >Ccrd_009859_ACYL2_GU248358MGSDQATIAVPNEHHKLMNINIKHSSFVQPSQPTPSSTIWTSNLDLVVGRIHILTVYFYRPNGASNFFDADVMKKALADVLVSFYPMAGRISRDQNGRLEINCNGEGVLFVEAESDSTLDDFGEFTPSPELRRLTPTVDYSGDISSYPLFFAQVTHFKCGGVALGCGVFHTLADGLSSIHFINTWSDMARGLSIAIPPFIDRTLLRAREPPTPTFDHIEYHAPPSMKTISQNPESSRKPSTTVLKLTLDQLNVLKASAKNDGSNTTYSTYEILAAHLWRCACKARGLPDDQLTKLYVATDGRSRLSPQLPPGYLGNVVFTTTPVAKSGDLTTQSLSNAASLIRTTLTKMDNNYLRSAIDYLEVQPDLSALIRGPSYFASPNLNINTWTRLPVHDADFGWGRPVFMGPACILYEGTIYVLPSPNNDRSMSLAVCLDADEQPLFEKFLYDF >Ccrd_001738MIMAKLQRFGRIRQLHTIISQETIKPSSPTPPHLKTHNLSLLDRFVGHIHMPIVFFYPNYDHGDTHILKKSLSQSLTQYYPFAGRFPAPHAPHINCNDEGVVFLEASNNGRLDEFIRKKEHDETMDRLIPNGLGCTMHKTSPNLIEVQLNHFAGGGAALAVSISHKLADALTMASFFNHWATVTRGGSPINPSFVSSSVTNNEILGFPLIDTEKLNYVRRRFVFPNSKLYELKNKVNAMGTSPMNPSRVELLTSLLFKCAVDSATRKSGSLMPSNLFHTVNMRNRNIKKFPETAAGNLSTTVIAKIATDSGEIKLHEVIGTLRKGIMELEELSNVEEVIGNLLSKLSPLEGEQSRAYISSSMCRFPFYEMDFGWGKPVDIMFRIPEVNDSCVLLMDAQSGDGIEALVRLQEEEMDIFRKDKDLLAYVEDM >Ccrd_000432MGIQGQSFSVKVVDKVVVSAEESWTDQWLSFTNLDLLVPPFNVSSFFCYNKPSYGSFPTMLNTLKASLSQALALYPPVSGDIAWNGAAGKNQIHCNNQGVDFTQAFADVELKELNFYNPDESIEGKLMPEKQRGVCATQVTELKCGGMVIAIKFDHRIVDGYSANLFISSWADLARSETPSMIPSFARSHMNPRSPPIYSSSIDDVFVPYVPQSQPDNDQNHDQGGDVLVNRIYYIESEQLKTLQSLASENGRRRSKLVAFTSFFWKKLALSMEDAGNHNEVCNVAVAVDGRRRLSEGDGEEKEKLMDSHFGNVLSMPYGTKKSQELNEMSLSNVATDVHEFLQTATGKDHFLDLIDWVEEQGPRPLISKAFAKGEMSVMVSAGQRFHTMDEMDFGWGKLAFGSCHVPSERKDCFVMTMGSPINNEDWVVYMHVPLKHLNFIEAHASHVFKPLNVDYLKI >Ccrd_023459MKCTDASCIQQQGGVINQYMADIHRGYNTSRGDCFVKPKILSEEAKKPIYFSPWDLPLFSFNYIQKGLLFRLPENQDFSIATFLDDLKDSLSATLTYFHPLAARLATVKQQKPPSLVAFLDHENSPGARFIHSTVDLRISDVLEPTDVPLIVQSFFDHHEAISHDGHQLSLLSIQVTELIDGIFIGCSINHMVADGTSYWQFFNSWSEVFRSKTQNGHLAPVSRPPILERLIPAGSDPIISLPFTDDDELIERYSPPFLRERIFHFSSDSLSKLKAKVNSDCNTTKISTLQGLSALVWRCVTRARQLPADQETGCRLAFLDPNSVHMGSSPRFDMYGNEFGLGKGVAVLSGFANKFDGKMTLYPGRDGGGSMDLEVCLLPENMAAFESDEEFLSVVS >Ccrd_025673MRVTIKPSSMVKPAEPTWSGRLALSELDQTGMTTHVPTIYFYTQPSDDWNTVLQTLITSLTTTLVHFYPLAGRLSSIAGGRLELDCNSAGVQLTEAYADIKLVDLDDLLESPMINKLIPSVDYRQTPLEDTPLLLLQVTRFCCGSWSLGFCISHVVVDGQSALHFLSEWARVCRGGLVASPPYLDRKILRAGESPITTCSSIHQYGQFIPPPILIGQSSNKNERRKKTTVAMMKLTETLVTKLRNKANQSRKNEGGHCFTRYEAVTAHTWRTACMIRNHESEQPTAIGICIDVRSKMKPPLPEKYFGNAIIDVIATGTSGEIVSKSLGYVSSKIKEAIEKVNDEYVSSMIDFLKNQEDLSKFQDLQWIRDDGGPFYGNPNLGVISWLTLPMHGVDFGWGKELFTGPGTGDAVDGDFLILRGEEASGSLVVASCLQVRHMEDFKRVFYQSIED >Ccrd_009741MGSCGRSYSLSVKQRDTIAAALPVQDHWLSMSNLDLLLPPLDVGVFFCYKKSPIPHEDSITTPVNLIKKSLAQALAPFYPFAGEVVQNSHGEPELLCNNRGVDFIHAQADVELKNIDLYHPDDSVERKLVTELNCGGLVIGCTFDHRIGDAYSINMFLTAWTEISRSRPISCLPSFRRSMVNPRRPPLMDTVYDSLFVPVSSLPPPRSYLPTNPLVSRIYYIQAKDIDQLQSNSSFNGNPKRSKLLSFIAFLWKIIAECDDGFETCKMGVVVDGRERLEQVNFDKLSLTNTKCFSMQNYFGNVLSIPYGEANSGELKEMALSQVAEMVYKFVSPAMTEEHFRGLIDWVELHRPEPAVAKIYTKTEETDGEAVVVSSGQRFPVESVDFGWGRPDFGSYHFPWGGQTGYVMPMPSVKKNGDWIVYMHLLEKHLDLVETKGRKVFKPLSPSYIGF >Ccrd_017486MAATDGFLTVLEICRISPPPNTVGERSLPLTFFDLGWLLFHPIHQIFFYEFPHSKSHFIQTVIPNLKHSLSITLQHFFPFAGNLIVFPPPNASGIARKPEIRHVEGDSIALTFAESTLDFNDLIGNHPRDCNKFYPLVPQLEGASKGSDFVKFPIFSVQVTIFPNSGFAIGLTNHHTLSDARTRYDLLRAWTSIAKYGTDELFLASGSLPFYDRVIEYPQLLDEIYFNQHVIQTLDENYRPPQLVSTTDKVRATFVLTRAHINLLKKWLLVQLPRLEYVSSFTLGCAYIWSCIIKSRLHLEGKKSEDELERFSCVIDWRSRLDPPVPQNYFGNCVGPCFATTKSTILTGNKGFVTAVELVGKTIRETVKNKQGMLKDAETWLDRFVIQVPTVGVAGTPKDDVYDLDFGWGKPKKIMQMTPSTTDMLTVLEDCRISPPPATVGERTLPLTFFDIMWLLHFPIHQLFFYELPHPKPHFIQTIVPIIRHSLSITLQHYFPFASNLIVFTDSNHSNVPKKPEIRHVEGDSVVLRIAESGLDFDDLVGNHPRACDKFYPLVPPLGRVAKVSNFLAIPLFAVQVTFFENRGISIGITNHHVLCDASTKYDFLKAWTSMARHGTDELLLAKGCLPSYDRPIKYPDSLDEIFMNQRGVETLNQEFQPPELVDLPSKVRATFILTKEKINLFKKWVLAELPTLGYISSFSVACAYIWSCIAKSRTEIHERKSEDELERFVCQANLRSRMDPPVPETYFGNCVGPCTAITKSMMLSGNKGFLIAVESLGKAISETVKNKEGVLKKAELCYEMLFITPEKISTKIGVAGTPKLKIYDVDFGWGKPKKYETISIDCNGSISLNAGKESPEDFEIGLSLPAKQMDAFVTIFNNGLDDSFSLSNYMDKLKSSSDNLLRDLHIPDYMLVPGGKVEALSVAPAYPTIVFINPKSGGQLGGELIVTYRSILGQNQVFNLEEEAPDDVLRRLYHHLENLKLNGDELAPLIQARLRIIVAGGDGTAAWLLGVVSDLQLSPPPPIATMSLGTGNNVPFAFGWGRRNPSTDSETVLQFLEQVKKAKEMEVDSWHILMRTKTTTEEGSCDPIPPLELPHSLHAFHRVSDTDEENISGYDTFRGGFWNYFSMGMDAQVSYAFHCERKLHPEKFTSQLANQVHRVHMQSLAVLKDGLQHLYFILLQSEIMNISQLAKIKIMKRNGRWEDLQISPRDLTPPYVDDGLLEIVGFRNAWHGLALLAPNGHGTRLAQCTYMRIDGEPWKQPLPVDDDDTIMVEISLHERVNMLATENCISKSVTDPCTPTTPCTPIDQDPEELDTDDSDSNCDEDFPTEQERRKFGACESFRLTEDLDIDHLTKFGACESFKLPDDLDIVHHL >Ccrd_013923MEVVVISKDSVKPSSPTPPHLKTFELSLLDQLVISPYVPIIFYYPNHHGHTILQAQEQSLALKRSLSETLTQFYPLAGTVKDDLSIDCNDVGAYYALASVRLRLDEFLSHPDLSLTDRLLPFRPSFEASGIGGRVTSVQVNIFECGGIAVGLCISHKIVDAASLYTFLKAWTNMARGSKEVVYPNLIGARSLFPAKDLWLKKASMAVCGSWLKEGLCVTKRFVFDADAISTLKAQATRNGVQNPTRVEVVSALLWKSAMAASKENCGFQKPSLLTHTVNLRRKLSSTLSKDLMGNLLWFTGADCQANDETTLDGLVKKVRDCVAKIDVEFANKAQGENGYIAMVESMKDMGEVSSKGTMDVYNFTSWCRMGFYDIDFGWGKPCWLTGVVGDGCPVFLNLITLTDTKCGEGIEAWVNMDEEEMKILESNSELLAFASLDPSPLPKDEVVAVNKPHAMNIEIDPINNYHGHNILQAQEQSLALKGSLSKSLTQFYSLAGTVKDDLSIDCNDVGAYYALALVRLRLNEFLSHQDLSSTDRRHIHTKAEATRNGVQNPTRVEVVTALIWKSAMAASKENCGFQKPSLLTHTFDAMDIEVISKESIKPSSPTPPHLKSFKLCVLDQLVVNPYVPIILYYPNSNGDSILQAQQKSLALKQSLSKTLTQFYPLAGTVKDDLSIDCNDVGAYYAIALVHLRLDEFLKQPDHSLINRFLPFQPSLEGSGAGARVTNVQVNVFECGGIAIGLCVSHKIVDAAATYTFLKGWTNMARGAKEVLYPNLNAPSLFPAKDLWLRESLMAMSSPWLKERVSCTTKRFVFDADAISTLKAEATRNGAQNPTRVEVVSALIWKCAMAASKTTCGFQKPSRFTLPVNLRRKLASTLSKDSICNVLWVTTADCPADSETTFDGLVKKVRKCISKIDIEFANKAQGDKGYAAMVESIKEMGEISSEGTMDNYLFASWCKMGFYDVDFGWGEPSWVSGIVGHGYPVVMNIINLIDTKCGEGIEAWCLMAMEIQVISKENIKPSSPTPPHLQTFELCLIDQIVIHPSYATLVLFYPNHNADTILQAQAKASALKDSLSKTLTHFYPLAGRIKDHCSIDCNDVGAYYALALAHGRLDEFLSLPDYTWMNSLLPFPSGFEPAGEDARITNIQVNIFKCGAIAIGLCISHKIVDGATVYTFLKGWSNMAFGGKEVVHPNLTAHSLFSATALWLREPLTAMWESWFKEGKCATRNFVFDAGAISALKAEATRNGVQNPTRVEVVSALLWKCAMEASKKAHGFKKPSRLTHAVNLRRKLPSAVSKDLVGNVIWLTSSEWRANHCTTLHDLVKKVHECIAKLGIEFVNKVQGDKGYVAIEESIKDRGEIGSKGTMDNYLISSWCRMGYYEIDFGWGKPSWVPGFIGHGSSVFANIVNLMDTRCGEGIEAWVFSTTVEKLGTVELDADKAMDMAHISKQIERMPKLLETP >Ccrd_000435MGISDMRFSVKVIDSVVVGAKEPWNDQWLPFTNLDLLVPPLDVGSFFCYHKPSHGSFPTMLNTLKASLSRALTLFYPLAGDIAWNAADRENQIHCNNQGVDFIQAFADVQLKELNFYDLDESIEGKLMPKKLRGVCAIQVTELKCGGMVIGIMFDHRIADGYSSNMFISSWADIARSETPSMLPSFQKSILNPRNPSTHSSSIDSNLFAHYDHRNRNDGPDDRIINRLYYIEGAQLNKIQSLASENGSRRSKLEAFTSFLWKTVAMSMEDLGNHNEMCTITLPVEGRRWLSEGDGEEKQKLMASHFGNVLSLPFGTKGSQELKEMSLTNIATEVHEFLQPVTRKDHFLDVIDWVEEHRSDPMVLPRALANKEMTVIVSSGQRFQFTDKMDFGCGKLAFGSCHLPPSRKDSYVMTLASPTNNEDWIVYMHMQIKHMNYIEAHASHIFKPLNADYLRI >Ccrd_020605MDIILSPPPPISPNLPLHEALEAQTTTKKRTNMKSFTLRSQFYIPTHSNLIANNPSFLISSKSKSSSLSVRKHKPTSQITKSPSRTNLFNRPLLFDSERVKITPKSNKSSSIEKLSGDEEPHPLGFSTKLEKLIPYGENSRYVIVGAVSMGFIMLLMGSDDHQKALAFGPEGPLMEDFWDNMRRYGLYALTVSSGVLYAVFQPLYELLKNPISAILVLTILGGGFYIVSQVVSAMDALLKRGKEHEDQPLIYPPSICADQLSLKQVSIILAVYLTLGTICFSLIQDQISGKKTNKILDAVYFCVITMTAAGYGDVGPETNLAKSLACVFVFTGMALGGFALSKAADYIVGKQEILFVKAIHMHETYGLNEILKETKTNKVKYKFLTILTLILFLMTIGTLVLSLVEKLSYFEAFYCVCATITTLGYGDKSFSSEGGRLFAIFWILITTVSLAQLFVYLVELWTENRRQVLVHWVVNRKLTIQDLEKADLDNDKVVSVAEYIVYKLREMGKVSDEDIAIVVEGFKSLDVDDSGTLTVNDIELMKSFEVYLFPFSFQHTHISTLLSQLLSTLVYHHSTMRWFLSAHKATRCFLISQQSHLHVASDLGSLHSMQMHSPSCFSAHQYTTSSYRSINGNKHEHTNTKKVDLEIISRETIKPAFPTPPHLRTFNLSIIDQVMFDCYIPLVLFLPNNNKATITHVVTKRSRHLKETLSRILTRFYPVAGEVKDDLHIECIDKGVYFVVARLNQTLEDFLGRPDEQKVRGLIPHNFGTLQSSKGNYLAGVQLNIFNCGGIGLCTSLAHKIFDGHTYFMFMKAWAAAVRGSPESFSPTFVASNLFPNNPSLKYSLPSKLMATESLSTKRFVFDSTALALLKAQPVSCTSSSVSRGPTRMEATTALIWKAAAKAASTIRPFSPQSPHALLSMVNLRGRASPPLPEECVGNIIYGAVGICFPESQPELATLMGEIRKSIAKINSEYIESLRGEKGQEVVNGTLKMLKDMTDGMHQGDCLIATSLLNSGIYEIDFGWGKPIWFYEMNAGYRLLALTDTIKGGGVEATVTLSSREMEIFERDPAVLSYATCNPSPLHH >Ccrd_018738KVIKTEVVAADQPWHDHWLPFTNLDLLVPPFDVGSFFCYKKSKDDDDGVTTMDMVDALKTSLSQTLVLFYPLAGEIVHNAAGEPEIHCNNRGVDFIQAAADVDLRDLNVYDPNESIDGKLMPPRHRGITTLKCGGMVIACMFDHRAADGYSASMFVSSWADMNRSVRPTSLPSFRRSVLNPRRPTLYSTSVANLFLPLSDLPPLLQTHPKSESPEPDQFDSHLISRIYYMEGEELKRLQFLASENGCQRSKLESFTSFLWKITASFLEESGHLDHMCKIAVAVDGRQRLSEGEGVEKQTLMATHFGNVLSIPFGGIRSKDLKDMPLSVVANQVHDFLKSAATKNHFLELIDWVEDQRPKALMSRPFARTEREFAIMVSSGQRLSIMGKMDFGWGKLAFCSCHVPSARTDCYVMTMGSPINDNDWVVYMHLPTKLLHYMETRASHVFKPLTSDYLKLSL >Ccrd_022243MKTAVMEKSREQVNVHSRLTVVSSTPTEPMGLACGLSPIDHIMGSHTGHIIFYYRTSPFLPKGRFSMDLDNFRVSLAELLSEYPRITGRLVRGHDGHGNWLVKYNDAGVRMFKAEVGVTVDEWLGFADESDERNLTVWEDMPDGDPTSWSPFQIQISEFVGGGLAVGLSFTHLLADPTAATQFYKAWTDAERGETIGNNPPVFNLPLLDSRPAPATTNGNTCTTTKYLQRHSKLVPTDPPMKMATSTFKFSNKMIEKLLSEIADKCPNATPFDYLTTLFWSRIIKLKTPASPSPIQSISLCIDARKLLDVPIPNKFFGNAISFSQLSLENEMLTGDSGLAEAVESVHRHVTGIKKDDILSTVDWLETCRNELNGMYPKPVQMYGPSLTCVSLEHLMIPKDEPKGEFESLVYEAKFRNNEKPVHVSYHVGKVEGEGLIVVGPSAEGGVARTVTVTLPAEEIGKLCRDPVIIEMEPAMILSGRRE >Ccrd_025215_HQT_DQ915589MTIGARDAQKMELTVKESLMVKPSKPTPNQRLWNSNLDLVVGRIHILTVYFYRPNGSSNFFDSGVLKKALADVLVSFFPMAGRLGNDGDGRVEINCNGEGVLFVEAEADCSIDDFGEITPSPELRKLAPTVDYSDQISSYPLCITQVTRFNCGGVSLGCGLHHTLSDGLSSLHFINAWSDKARGLSVAIPPFVDRSLLRARDPPTAMFEHLEYHSPPSLIAPSQNQNSTSHPKLASTTMLRLTLDQINGLKSKAKGDGSVYHSTYEILAAHLWRCACKARGLSDDQPTKLYVATDGRSRLNPPLPPGYLGNVVFTATPIAKSGEFKSESLADTARRIHIELAKMDDQYLRSAIDYLELQPDLTALVRGPTYFASPNLNINSWTRLPIYESDFGWGRPIFMGPASILYEGTIYIIPSPSGDRSVSLAVCLDPDHMSLFRKCLYDF >Ccrd_005848MAIDIVKQSSKFIKPATPTPPNLRHFRIGFIDEFAPSANVSVVLFFSINGDRNPKFIAQLGESLQTTLIRFYPLAGRYAAETRTIDCNDQGAEFMHAKVNIKLEAFLDSEVNVKLVDKFLPREIGESGLITDPILAIQVTTFECGGVALGVSISHKIADASTLSTFLNEWAVINREEKRIELTGSCRFNAASLFPSRGVPALDLGFSRSIRDDDMQSKYVTKKLSFSESEISNLKEKAMLINGRNGTPQWSKVQLVSAIILKSFICVDRAIRDDPRDTILIHPINLREKTASLIPKDSCGNLWGILATECGVVEATEALADLLRDSIKNTISNYLKVSHDDEGEQTMVLNSILNVGQIPQTANVIWMSSWCKFPFYEVDFGFGKPIWANPGGMPVKNSACLMDGVGGDGVEAYVCMEPKDVPYFEESLEIKVCVG >Ccrd_022067MEAKLSNIRLSTVVPGQMTVENTVHHLTNTDLAMKHHYIKAVYFFNNEFAELGLIISDLKTAMSHLLSRYFTVAGRIRRRPEDNRPFIKCNDGGVRVVEAESNTSIDEWVEMKHSDVSELAYRDHVLGPDLQFSPLLFLQFTKFKCGGFSVGLNWSHVIGDAFSTSTFVNMWAQIVNCKTLSHISISTKLQISERPSTFQNIDLVGNHWFNVNNTKMVTFSLHISSKNLDQITSKANAKPFQALCATIWKSLAKIRAQLEPKTVTICTYGSQKNEIKSQGNNQRVSKVVVDFTVSEASVIDLAELITVKQNNSDAMFYGETLTFVNLEEAKLYGFELKGHKPVLANYAMTGVGDEGVALVLQGPENYRGRLLTMTLPSDQVVALKNELIKEWNIGRD >Ccrd_020450MAPFSSSWVQERHFPDLLIPISIDRAFSVLPEGPIQAQDGDTLYLSNLDDIIGARVFTPTVYFYTATRSSFDDVVDILKDALGKVLVPYYPFSGRLRGTNNGKLEVFFGPNQGALMVQAHTELSLDRLGDLTVPNPAWSNLVYKFPNEGNYKVIDMPLLIAQVTRFSCGGFSLGLRICHCICDGLGAMQFLGAWASTAKTGSLVVDPNPCWDREIFVPRDPPMVKYPHVEFMKIDDGSNLTISLWEVKPLQKCYWLSREFQAHLKSVARPMDSLGCTTFDAMAAHVWRSWVKALNVKPLDFGLRLTFSVNARSKLKNPPLKDGFYGNVVCVACATCTVSNLINGSLQDVTRLVREARLGVSEEYLRSTIDFVEVDRPNKLEFGGKLTITQWTRFSMYESSDFGWGRAIYVGPIDLTPTPQVCVLLPEGVADSSGAMVVCICLPEAAAHRFKELLCLMDT >Ccrd_014640MPSSQPLVHIISKSTLFPSTKSTLSHLKLSVSDLPMLSCHYIQKGNLFNRPPISISNSDLLFLLKCGLSRALTHFPPLAGRLTTDSDGYVYITCNDAGVEFVHANAVHLSITDITSPLHVPDSVKGFFAFDRTVSYDGHFKPILAVQVTELKDGVFIGFSVNHAVVDGTSLWNFINTYAEVCRGAKLVSKQPSFVRDSVLISSACLRLPAGGPKVTFDENAPLSERIFSFSRESILKLKDRTNNRKRMIFYGNSEINTDELLGKQRNDRITHSDDIGNWIRSDVALKPEPVPEISSFQSLCALLWRAVTRARKFPNSKTTTFRMAVNCRHRLKPELETFYFGNAIQSIPTYATAGDVLSHDLKWCAEQLNKNVLSHDHTMVCGYVKNWESDPRCFPLGNFDGAMLTMGSSPRFPMFDNDFGWGKPVAVRSGRANKFDGKISAFPGREGGGSVDLEVVLSPETMAGIESDPEFMQYVWDKC >Ccrd_021735MGNKNPAXVIRDALAKVLVYYYPFAGRLXEAPTQKLMVDCTGEGVLFIEAEADVTLKHFGVSLHPPFPCLEELLYDVPGSNGVVDSPLLLIQVTRLLCGGFIFAYRINHTMCDGPGVXQFLTALGEMAQGASSPSVLPVWQRELLFASEPPRATFAHHXYDVVGKTKAINDIVTKDLIQKSFFFGPNDVSTLRRRFVPEHLQRCSTFEVIAACVWRCRTIALQFDPNEEMSFLFPFNIRDKLNPSLPVGYYGNAFILPSVVSTAGDLSIKPLSHVLELVRKAKSLVSEEYVRSTIDLMAIKGRPLLTVPRSYILSNVARPGFSEIDFGWGKAAYGGPATGGLDAIPGLFYFYTHSTNEKGESGVVNEFAFAFSLS >Ccrd_021736MAQINTPLTFAVRRRAPELIVPAEPTPRELKPLSDIDDQEGLXFQIPVIQFYRRDPKMRNKNPATVIREALAKVLVFYYPFAGRLKEGPAXKLMVDCSGDGVLFIEAEADVTLKQFGDALQPPFPCLEELLYDVPGSGGVLDSPLLLIQVTRLLCGGFIFALRLNHTMSDAPGLVQFMTGLGEMAQGASRPSTLPVWQRELLFARDPPLVTCIHHEYDVVEDTKGTIIALDDMAHRSFFFGPAEVAAFRRFVPTNLQKCSTFEVLTACLWRCRTIALQPDPEEEMRIICIVNARAKFDPPLPKGYYGNGFAFPVAISTARDLTNKPLGHALEQVMKAKNDVNAEYMRSLADLMVLKGRPHFAVVRSFLVSDVTRAGFDEVDFGWGKAAYGGPAKGGVGAIPGVASFYIPFINHKGESGIVVPVCLPSAAMKIFVEELNTMLMPNKKAQVRQEHDQFALSRL >Ccrd_014129MGTLYQSPTISTSPKIIQDLKVTIHDSITIFPPQETKTEAGSMFLSNIDQVLNFNVETVHFFVANPQFPPPLVAEKLKSALSKALVPYGFLAGRLRLNPESRRFEFDCNGAGAQFVVGSSEFELGEIGNLVYPNPGFKQLVVKSYDNLDIVDRPLCILQCGGFAIGVATNHATFDGISFKIFLENLASLAADKPLATAPCNDRHLLAARSPPHVQFDHPELLKIPEGIDLPNPTVFDCQDELDFKIFNLTSKDITHLKQKAKDEPIPTRAKVTGFNVVAAHVWRCKALSSGTDYDPDRVSTILYAVDIRSRLNLPHSFAGNAVLSAYASAPCKEIEKSPFSKLVELMTEGASRMTGEYARSVIDWGEVNKGFPAGEFLISSWWRLGFTDVEYPWGKPRYSCPVVYHRKDIILLFPDIVGESDNEVNVLVALPSKEMAKFEALFHKFLA >Ccrd_018370MPSSSSSSSSGSVAIISKCTVYPAKNSITKSLKLSVSDLPMLSCQYIQKGVLLSQPPMPFNHLISLLKLSLSKTLSHFSAIAGRLSTDPQGHVHILCNDSGVEFIHATATHHFTDQILLPNCDVHPCFKTFFAFDKTLSYAGHHQPIAAVQVTELGDGVFIGCTVNHAVVDGTSFWNFFNTFAEISKGAKKVTNSPDFSRENVFISPVVLPLPAGGPSATFSGDEPVRERIIHFTREAILKMKFRANNQIWNPQNSDSNESEIYGKASNDVNGKLNGALKPKSEISSFQSLSAQLWRAVTRARKFPETKTTTFRMAVNCRHRIDPKVDPHYFGNLIQSIPTVASVADLLSHDLSWAANQLHQNVVAHDNAIVRRGVKDWESNPKLFPLGNFDGAMITMGSSPRFPMYNNDFGWGRPLAVRSGKANKFDGKISAFPGRDGDGSIDLEVVLAPETMAALEHDQEFMQYVS >Ccrd_023461MKSPSVEIISDVFIKPKLVSEEAKKPIYFSPWDLLLFNIHYIQKGLLFRLPENLDFSIATFLEDLKDSLSVTLTHFHPLAARFATVKRQNPPSLVLFLNHENSPGARFIHSTVDLRVADVIEPTDVPLIVQSFFDHHDVIAHDGHQLSLLSIQVTELIDGIFIGCSINHMVVDGTSYWHFFNSWSEVFRSKTQNGHLTPVSRPPILERWIPAGSDPIISLPFADDCELIDRYRQPFLRERIFHFSSNSLSKLKAKVNSDCNTTKISTLQGLSALVWRCVTRARRSPADQETGCRLAVNNRSRILPPVSESYFGNMVSIVTGKTTAGELLDQSIGRAAWRLHEAVVNHGDKAIKGFVDSWLKKPFVFKLSQFLDPNVVHMGSSPRFDMYGNEFGLGKGVAVMSGYANKFDGKMTLYPGREGGGSMDLEVCLLPENMAAFECDDEFMSVVNGEKPI >Ccrd_022272MTSSPTVTRISECFVKPPDDHLSPEEKQPIHFTPFEILSLNIKYSQKGLLFAKPPPSENQDFSIPVFLDHLRRSLSAALTHFYPFAARLATRIQENPPSYVIYLDPENSLGAKFVYATVDATVSDILTPPDVPLVVRSFFDLNNAINHDGHTLPLLSIQVTELVDGIFIGGSVNHLIADGTSFWQFMAVWSENFRSKDRASISHPPVHRRWDESDPIINLPYTHHDQFIERFEPPPFKERFYHFSSPSVSKLKSKANSECNTDKISSLQAVIALIWRCITRARRLPPEDQTSCRLMVSNRRKMNPPLSDYYLGSPVQVVRATATVEDLMAHGLGWAALRLHEVVMNHDHSKVKEMVESWIKRPVIFKMSHGIDRNAIHVGSSPRFDMYGCEFGLGKAVAARSGGANKGEGKITMYPGREGGGSMDVEVCFGSELMMDMECDEELMSALMVD >Ccrd_007826MPMVVFYPNSAIHAKTLDLKNSLSQTLTQYYSFAGRHAKIASAYVDCNDEGAEFLEASVNGTLSNFLQNSLHEDLDQFFPYGTVGTVKVMIFEATE >Ccrd_000433MGIMGKSFSVKVVDKVVVSAEEPWTDHWLSFTNLDLLVPPFNVSSFFCYNKPSHGSFPTMLNTLKASLSQALALYPPVSGDIAWNGAAGKNQIHCNNQGVDFILAFADVELKELNFYNPDDSIEGKLMPEKQRGVCAIQVTELKCGGMVIAIKFDHRIVDGYSANMFISSWADMARSEAPSMIPSFTRSHMNPRSPPIYSSSIDDVFAVYSPQSHPDDDQNHDHGDNIAVNRIYYIKGEQLKRLQSLASESGRRRSKLVAFTSYLWKNLALSMEDAGNHNEVCNVAVAVDGRRRLSEGDGEQKEKLMDLHFGNVLSIPYGTKKSQELNEMSLSNVATDVHEFLQTATGKDHFLDLIDWVEEQGPQPLIAKAFASKDMSVMVSSGQRFHTMDEMDFGWGKLLFGSCHVPSERKDCFVMTMGSPTNNEDWVVYLHLPLKHMKFMEAHASDVFKPLDADYLKI >Ccrd_011701MPCESVMEFSATQIISKSTVFPATKSTLSDLKLSASDLPMLSCHYIQKGNLFPRPSIAITDLLLLLQQGLSRALAHFPPLAGRLITDANGYVYITCNDAGAQFVHASATHLTVNDVISPTHVPDSVKGFFSFDRMVSYDGHFNPILAVQVTELDDGVFIGFSVNHAVVDGTSLWNFINTFAEVCRGANLISKQPFFTRESVLISPAVLRVPADGLKVTFDEYAPLSERVFSFSRESILKLKDRTNNRKKFSCYGNGEINAAEVMGKQSNDPIKLSDEKVTTLIGNWIRNAVVTKTEPAREISSFQSLCALLWRGVTRARKFPNSKTTTFRMAVNCRHRLEPKLETLYFGNAIQSIPTYATAGDVLSHDLKWCAEQLNKNVLSHDDTMVRRAVHSWEQDPRCFPLGNFDGAMLTMGSSPRFPMFDNDFGWGKPVAVRSGRANKFDGKISAFPGKEGGGSVDLEVVLSPETMAALELDPEFMQYVSGQCC >Ccrd_017478MASLPILTVLDHSHVSPPPATVADASLPLTFFDILWLTFSPVHHLFFYELPSVSKTHFIEDIVPNLKQSLSITLQHFFPFTGNLIVFSSPTRKPEIRYVDGDSVAFTIAESALDFDDLTGNHPRDCGKFYHLIPLLPEATKESDHVKIPVFSVQVTFFPNSGISIGMTNHHSLGDASTRFCFLKAWTSIARSGSDESFLANGTLPIFDRVVNYPKIDELYLKNLEIETTFNKDYQPPRLSAPTDKVRATFILTRTVINRLKKWVSTELPTLPYVSSFTVLCAYIWSCIAKSRNDELEIFGFSVDCRARIDPPIPAAYFGNCVALCMSIAKTDLLTGNDGFLNAAKLHGENLHKMLTDKDGVVNDKWPFGGLLSQGTPTTMMGVAGTPKLKFYELDYGWGKPRKHETISIDYNDSISLSACKESNEDLEIGVCLSAIEMEAFIPIFNKGLEACF >Ccrd_013924MEVEIISKETIKPSSPTPHHLKTFKLSLLDQIVLNPYLPVIFYYQNRNGDTIFQAQEKSSSLKESLSKTLTQFYPFAGTIKDDISIDCNDAGAYYAIALVRLRLDKFLHDPDLKLTDGLLPFRPTFEASGPGARVTNVQVNIFECGGIAIGLCMSHKIVDAAALYTFLKGWTNMACGATEVVHPNLTAPSLFPANDLWLREASMEVCVSWLKEGKCSTKRFLFDSDAISSLKAEATRNGVQNPTRVEVVTALLWKCAVAASKQTCGFQKPSRINHTVNIRRKLASPSSKDLIGNVIWFATAECPANDETTLIDLAKRVRECVSKVDVEFVNNAQGDKGYIAMREFMKETGETSSKGSTDVFNFTSWCKMGFYDIDFGWGKPSWMTGVIGIGNPVFLNIINLMDTKCGEGIEAWVNLDEKEMEILQCNPELLAYASLDPSPGLSS >Ccrd_000436MGKSFSVKVIEKVVVGAEEPWNDRWLPFTNLDVVLPTFDAASFFCYNKPSHGSSFPTMLNTLKASLSQALTLFYPLAGDIQWNAAAGENQIHCNNQGVDFTHAVADVQLKELDFYNPDESIEGKLVPKKLRGVLAVQVTELKCGGMVIGCMFEHKTADGYSANMFISAWADMARAKTPSMLPSFSRSVLKPRSPPTYSCSSVFEHIVATLEPSLMPDNDENQNDDDQLINRVYYMEGEQINKIQLLASENGSKRSKFEAFISFLWKAIGMSMEELGNEKERYCNVAIPVDGRRRLSEGDGEEKQKLMASHFGNVVTLPFGGKGSQMPLSNVATEVHELLHTVTGKDHFVDLIDWVEERRSHLLIPRAFVNKDMTVIVSSGQRFQFMNEMDFGWGKVAFGSCHIPPTRKDCYVMTLPGPITNNQDWVVYMHLPIKHINYIEAHASHVFKPVNADYLNI >Ccrd_017480MPPVHNLYFYELPAVTKTHFTETIVPNLKQSLSITLQHFFPFSGNLIVFPTPARKPEIRYVDGDSSTLDFNDLTGNHPRDCDKFYHLIPILPDSAKESDHVKILVFSVQVTFFPNSGISIGMTNHHSLCDASTRFCFLKAWTSIARTGSDESFLANGTLCFFDRVVNYPKIDEIYLKNLEVETTFNKEYQLPRLCGPTDKVRATFILTRTVLNRMKKWVSTQLRTLPYVSSFTVACGYIWSCIAKSKNDELQIFGFTIDCRARTDPPIPAASFGNCVTLCTAIAKTPILTGKEGFLTATKLLGENLHKILTDKDLEIQWYQIWWYILLSSLTKKTMI >Ccrd_006170MTMSWNSYVRTPIPHAPFTKIHRVNVFMAMKTEIQSRKLIKPSISTPPSLRSYKISFVDELAPSMNISLVLFFPHNTDYNPTQLEKSLEKTLTLMYPLAGRYMADVRTVDCNDQGVEFVQAQADTTIQEILDFKLKVDPNLINQFIPSKLLADGPTDAVLATQLTAFECGGSALGVSIAHRIGDVSTMSAFLNQWATLSRKDAKVETGLITSFSSFPAQDSPFIEQGFTKLNDNGYVTKKLSFDENAISNMRMKATSNGKTDNRQLFKVQLVSALIWKAFIGVDHAIYGHSRDSVALQPTKLRQKTRSSLPRSISIGNQWGPIVTERNTTKKVELGFEDLIDLLGDSVMKTMKEYSKLGHDDSQERKEMVLKSFSQIRNISNDRNVVWLVSWCRFPYYDVDFGFGKPEWISCGCVPFKRGVIMVDDAGGNGVEAYVSMGVADVRHFEQDEDIKAFSV >Ccrd_011676MTNMESSAAQVHVKEAALVVPSDPTPTHLLKLSSVDSQLFLRFTIEYLLIYRFNGLHTDHVTARVKSALARALVPYYPLAGRVRVRPDGSCLEVVCRAQGAVFIEATADFTLSDFERAPRYVTEWRRLLALQVADVLKGAPPLVVQLTWLSDGAAALGVGYSHCICDGVGSVEFLNLFAALATGRRHGGGLEFKPKPIWQRHLLDQTPFKQPRPRQHLEFSRVTDHCQFMTRFTPDQLTPTAVTFDEWRLNELKNSITPTSQLSKSSLTSFEVLSAKVWRSWAKALNFPPQQILKLLFSIDIRHRVKPSLPTGYYGNAIVLGCAQATARDLTEKGLAYATELIKEAKNRVDDQYVKEVVNSVSLNGARGVPDPVGVLILSQWSKLGLERVDFGIGRPVQVGPVCTDKYCILLPVDDHSRSVKVMLAVPSVAVDKYVDLMRAVQ >Ccrd_021400MVSSDKPNPVYDIRISTVAPGFISGHDAAQELSTMDLAMKLHYLRFVFYFPSPAFDGFTILNIKESMFNWLSHAYIPCGRFRRSDSGRPYIKCNDSGVRIIEAKCHLGLDEWLESRDDSRNTLLVPNNVIDAFSAMGFIKLWAQAIVGHYPAQPLTMAQPQVHARNCQSLNPSPDPLSVKRVDPVGDLWSTSNTSKLETFSLCISTSELIRLQAKICREKGERKISPFECICVVIWQCVAKARQGLGPEAVTICRSDMRNRAKGIITNKSQTISLVKTDLSVAKSDPMELGLLIMNQAVDERMKIEETIERDDELPDFLIYGANLTFVDLSDVPFYEMEVRGQKPAYVNYAIDNIGDEGVVLVLPTPKNCPDGKMVSVTLPENQVVELKSTLKEDWCIA >Ccrd_021080MAMPFHVNILEKCHVSPPPDSIIPPISLPLTFFDIPWLLHPSNQTLFFFPKPPAKSSTTTVISLLKQALSLTLHHFHPLAGNLSAPPPPAEPHIVYNKGDSVSLTIAESNANISHLSGNHPRSITMLYSLLPKLPFPSKSRDTHVVLVLPLLAIQITVFRDLGFSVGVTAQHAAADERTLDQFIKCWASVCKSLLKKDSFFAFKPQPFFDRTIILDPNSHKTTFLKQWWHRRLMNSPKDSHQEIIGHNIVQATFILSSSDINMIKHHILAKCKTIKEDPPVHLSPYVSACSFIWVCLLKSEEETYDSKGTTTPLYIGFNAGGITRLGYEIPSSYFGNCIAFGRCKALGSQLLGEDGVVFAAKSIGKEIKRLDKDVLEGAERWICEWDELNIRVLGSPKVDSYAMDFGWGKVDKVEKLSSDDHHGRVNHVISLTGSRDLKDGMEIGVVLSRATMNAFTRLFSGGLLELAIS >Ccrd_026144MEIEIISKESIKPSSPTPHHLKTFELSLLDQLVVDPYVPIVLYYPNHNGSNVLQALERSLALKKSLSKTLTQFYPLAGTIKNGLSIDCNDVGACYAIALVRCGLNELLSHPDHQLLNGLLPFQPSFEGSSVGARVTNVQVNIFECGGIAIGLCISHRIVDGAALRTFLEGWTNMACGAKEVVYPNLAAPSLFPAKDSWLRDSSMAMCGSWLKEGKCVTKRFVFDSDSIARLKAEATKNGVKNPTRVEVVSALIWKSAMAASKQITGFQKPSRLTHLVNLRRKLSATLSKDSIGNVIWPATAKFQANYETTLHGLVNKVRESISKIDIEFVNKAQGEKGCVAMQESIKEMGEISSKGTMDNYTFTSWCKMGFYQMDFGWGVPSWVSGIIGHGSPVFINQVTLMDTVFGEGIEAWVNLDEEEMEILQGNSELQAYASMDPSPLPKLTE >Ccrd_017488MASLPIFTVLEQSQVSPPPATVGHRSLPLTFFDIGWLSQPPVHHLFFYELAITKPQFIETIVPTLKRSLSITLQHFFPFVGNLIIFPTRKPEIRHVDGDSVAVTFAESNLDFHDLTGNHARDCGSFYPLIPVLGHAAQASADYVSITVFSVQVTVFPGCGISIGMTNHHCLGDASTRSCFLKAWTSIARSGTDESFLGSGALPLYDRVIKQPTLDEIYLKQANIGTFTQEYQPASLSGPTNNVRATFVLTRPTINRLKKRVSNQLPSLQYVSSFTVACAYIWSCMAKIRGDELQVFGFVIDCRSRLVPAIPATYFGNCVAPCGAMARTTVLSERDGFVTAARLLGESLHEKLTDKDGILKDAESWYELSFGGVPDTIMGVAGTPKIRFYDTDFGWGKPRKYEIISIDYNGSISMNACKDSNEDLEIGVCLSATEMKAFVSMFNCGLE >Ccrd_020279MSKYSHLLTIGRSKVLSILNNKWVHESCLIRSYHPQPPFATLNHHHLHNSCSRVSTVSHYYHNHHHHSQLSTIHTLELPKHEGDSPLDYQVTVKDRDIISASCAPLHEYWLPQSNLDLLLPPLEAGVFFCYKKKDDTVMSPGTVVKTIKKSLGRVLSTFYPLAGEIVSNSQGEPEVLCNNCGVEFVHAHADVELKTLDLHHPDENVSGKLVPKINRGVTELKCGAIIVSCAFDHRVADGYSLNMFLVAWAEFSQFKQISITPSFRRSILDPRRPPRYNAIFDNMYLPLSSLPPPHPCEHQLHSRIYYIKKESLNRLQSEANSKETRISKFQSLTAFIWKLLAHQADNDVNRTSRMGVVVSGRRFLTGNSEKESSMLENHFGNILSIPYGEMNNCCLQMMPLNEVADKVHSFVTKATTEEHFRGLVDWVELHRPEPAVAKIYFKLHETDGEAIVASSGQGLPIKDMNFGWGKPEFGSYHFPWGGQTGYIITMPSASKNGDWVVYMHLKQKHLDLIEMNTPHIFNPLTYDYLSFH >Ccrd_006312MTSPPVKLISECYIKPXDDSLSVDANQPIHFTPFELPFLNANYSQKGLLFAKPPPENQDFSVTTYLDELRXSLSATLTQFYPLAARLATRKEKNPPSYVIYIDPENSPGVKFVYATVDLNISDIIMSTHVPSVVHSFFDLNNVXNHDGHTLPLLSIQVTELNDGIFIGGSINHLIADGTSFWHFMAAWSETFRSKDQKRCFRRSAIQGYKPIINLPFTHHDQFIERFEHPQFKERFFRFSSATVSRLKEKANAECNTHKISSLQAVTALLWRCVTRARRQSSDSETICKLVINNRRRLNPPLSDDYFGNPIQTVRGTARVEDLMAHGLGWAALRLQQAVANHDHTAVEKSVESWLKKPVIYKLSDLFHSNVVHIGSSPRFDMFGCEFGLGKAVVARSGGANKADGKMTMYPGRNGGGSMEVEACLLPEFMEDLEDDEEFMNALSDHN >Ccrd_017481MSHEMTNSLPILSVLEHSQVSPPPATVGDNSLPLTFFDIYWLNSPPVHHLFFYDLPLTKARFTEIIVPNLKRSLSITLQHFFPFAGNLIIFPTPTRKPEIRYGEGDSIAVTVAECNLDFDDLTGNHPRDCDKFYHLIPSLGPAVKVSDYVSIPVFSIQVTLFPNHGISIGMTNLHALGDATTRFGFLKAWTKIARSGTDESFLANGTLPLYDRVVQNQKLDESYLKRVNVEAFNEEYRPQNLSGPTDKVRATLVLTRTTINRMKKRVSTNLPTLEYVSSFTVACGYIWSCIAKSRNDELELFGFAVDCRARMDPPIPAAYFGNCVGGCAVMAKRTLLTGKDGFVTAAKLLGENLHELLTHKDGIVKDIFAMIDDLLSNGMPTTTMGVAGTPKLKFYDIDFGWGKPKKHETISIDYNNSISLSTCKESKEDLEIGVCLSAMEMEAFVHIFHEGLEAYL >Ccrd_022378METKSVVVMKKKMPTPPENKLHFATSQSIFLPDPKKLASCFLSVDANQPIHFTPFELPFLNANYSQKGLLFAKPPPENQDFSVTTYLDELRXSLSATLTQFYPLAARLATRKEKNPPSYVIYIDPENSPGVKFVYATVDLNISDIIMSTHVPSVVHSFFDLNNVINHDGHTLPLLSIQVTELNDGIFIGGSINHLIADGTSFWHFMAAWSETFRSKEQKRCFRRSAIQGYKPIIKLPFTHLDQFIERFERPQFKERFFHFSSATVSRLKEKANAECNTHKISSLQAVTALLWRCVTRVRRQSSDSETICKLVINNRRRLNPPLSDDYFGNPIQTVRGTARVEDLMAHGLGWAALQLQQAVANHDNTAVEKSVESWCKKPVIYKLSELFHSNVVHIGSSPRFDMFGCEFGLGKAVAARSGGANKADGKMTMYPGRNGGGSMEVEACLLPESMKDLEDDEEFVNAVSDHN >Ccrd_021731MLQPETSLKFVVRRRAPELIAPAAPTPRELNPLSDLDHQPELRCQLHGLQFYRSHPKMANKNPATVIRDALAKVLVYYYPFAGRLREAPTQKLMVDCTGEGVIQFLTAFGEMAQGASSPSVLPVWQRELLFASEPPRATFAHHEHDVEEKTKAINDIGTKDLIQKSFFFGPNEVSTLRRRFVPEHLQRCSTFEVIAACVWRCRTIALQFDPNEEMRFLFPFNARDKLNPRLPVGYYGNAFILPSVVSTAGDLSIKPLSHVLELVTKAKALVSEGYVRSTIDLMAIKGRPLLTVPRSYILSNVARLGFSEIDFGWGKAAYGGPATGGIDAIPGLFNFYMHWTNQKGESGVLNFTFHNLTS >Ccrd_004326_ACYL1_GU248357MGSDQKMMMNIDIMKSSIVPPSELIADCPKQLWTSNLDLVVGRIHILTVYFYRPNGSSKFFDPNVMKKALADVLVSFYPMAGRLGRDETDRIVINCNNEGVLFVEAESDSTLDDFGELKPSPEFRQLTPSVDYSGDISSYPLFFAQVTHFKCGGVALGCGVHHTLSDGLSSLHFINTWSDMARGLSVAIPPFIERTLLRAREPPTPTYDHVEYHSPPSMNTTAQKPGSGSLSKSSTTMLKLTLDQLNSLKAKAKSESGSTHSTYEILAAHIWRCACKARGLPDDQLSKLYVATDGRSRLSPRLPPGYLGNVVFTATPVAKSGDLTSKSLSNTAKLIHTTLTKMDDDYLRSAIDYLESQPDLSALIRGPSYFASPNLNINAWTRLPVYDADFGWGRPIFMGPACILYEGTIYVLPSPNNDRSVSLAVCLDANEQPLFEKFLYEF >Ccrd_014329MMVKVEIVSKENIKPSSPTPQTLKSFKLSILDQLIPAPYAPIILFYPNQDHGDGATDLDHQLHTRLETLKDSLSKTLTDFYPLAGTIKDDLYIDCDDVGAYFVVARVNTRLRDFLENPDLELVNHFFPCAPGFNGSVAGCCVTNVQVNVFECHGIAIALCISHKILDGGALSTFLRGWTGSSRGSKDVVIPNLGAPSLFPANDLWLQDSAMVMWGSLLKFGKCSTRRFVFDSSKLAVLKAEAAGNGVKDPTRVEVVSALLWKCAMAAAEEKVGFRKPSMLSHVVNLRKRLASTLSEDSIGNLIWITSSECGPESEIRINDLVERVRGSVSKINGEFVKNIRGDKGREVMEESLEKLKDCGTTKDYIGFTSWCKMGFYEADFGWGKPIWVCGSVSDGSPVFMNFVVLMDMRFGDGIEAWVNMDEQEMEILQHNQELMAFASLDPSPLQTNQLSVF >Ccrd_026842MEVEVISKECVKPSSPTPPHLKTFELSLLDQLHIIPYVPVIFYYRIHNGHNILQAQEKSLALKGSLSEILTQFYPLAGTVKDDLSIDCNDVGAYYALALVRLRLNEFLCHPDLSLTDRLLRFRPSFEASGIGGRVTSVQVNIFECGGIAIGLCISHKIVDAATLYTFLKAWTNMACGSKEVVYPNLIGSRSLFPAKDLWLKEAMAVSGSWLKEGIWCKMGFYDIDFGWGKPCWVTGVIGDGCPVFLNLIXLMDTKCGEGIEAWVNMDEEEMKILQSNSELLAFASLDPSPLPKDEVVALNKPHAMNTEIDPIK >Ccrd_023462MASPTVEIVSHCFVKPKFPSNKPIYFSPWDLVMVNVNYIQKGLLFRLTENQDFSIVTFLEDLKDSLSATLTHFHPLAARLATVKQQNPPSLVVFLNPENSPGARFIHSTVDLTVSDVLGPTDVPLIVQSFFDHHEAIDHDGHELSLLSIQVTELVDGIFIGCSINHMVVDGTSYWHFFNSWSEVFQSKTQNGNIAPISRPPVLERWIPTGSDPVLSLPFTHNDEFLDRPNRPFLRERIFHFSSESLSKFKAKVNSECNTTKISTLQCLSALMWRCVTRARRAPENKETGCRLAVNNRSRLSPPLPDNYFGNSIQTVRVVTTAGVLLDQSLGWAAWMLHELVMNHGDKAIKEFVGSWVKRPFVYKMSQLFDGDSIQMGSSPRFDIYGNEFGLGKGVAVLSGYANKFDGKVTLYPGRDGGGSVDLEVCLLPENMAAFESDKEFMSVVNGEEAIVTHKIAFLRHELEMATKSELSRSQFEHIHPQRKVERTLQKALLDGFHDILASGTKDSVEINPPL >Ccrd_001737MIMAKLQRFGRIRQLHTIISQETIKPSSPTPPHLKTHNLSLLDRFVGHIHMPIVFFYPNYDHGDTHILKKSLSQSLTQYYPFAGRFPAPHAPHINCNDEGVVFLEASNNGRLDEFIRKKEHDETMDRLIPNGLGCTMHKTSPNLIEVQLNHFAGGGAALAVSISHKLADALTMASFFNHWATVTRGGSPINPSFVSSSVTNNEILGFPLIDTEKLNYVRRRFVFPNSKLYELKNKVNAMGTSPMNPSRVELLTSLLFKCAVDSATRKSGSLMPSNLFHTVNMRNRNIKKFPETAAGNLSTTVIAKIATDSGEIKLHEVIGTLRKGIMELEELSNVEEVIGNLLSKLSPLEGEQSRAYISSSMCRFPFYEMDFGWGKPVDIMFRIPEVNDSCVLLMDAQSGDGIEALVRLQEEEMDIFRKDKDLLAYVEDM >Ccrd_017487MAATDGLLTVLEKCRISPPPNTVGERSLPLTFFDLRWLLFHPIHQLFFYDFPHSKSHFVQTVIPNLKHSLSVTLQHFFPFASNLIVFPAPNPSGLARKPEIRHVEGDSIALTFSESTLDFNDLIGNHPRDCNKFYPLVPQLEGASKVSDFVKIPIFSVQVTIFPNIGITIGLTNHHTLSDASSRYDLLRAWTSIAKYGTDEMFLAGGSLPFYDRVIEYPQILDEMYLKLPPIQKLDEKYRPPALVSQTDRVRATFILTRAHINLLKKWLSVRLPTLEYVSSFTLGCAYAWSCIAKSRLHLEGKTGENEQERFVCVIDWRSRLDPPVPQTYFGNCVGPCFTTTNSTLLTGNKGFVTAVELVGKTIRETLKNKQGMLKDAETWLDRSILQVPTVGVAGTPKLNIYDVDFGWGKPRKYETISIDFSRSISVNVSSESAEDLEFGVSLPAKQMDYFSTIYGQELEDIISEEI >Ccrd_017471MASLPILTILEQSHVSPPPATVTDASLPLAFFDILWLPFSPVHHVFFYELPAASKTHFTETIVPSLKQSLSITLQHFFPFSGNLIVFSTPTLKPEIRYVNGDSVAFTVAESTLDFNDLTGNHPRDCGKFYHLIPLLPEATKESDHVKIPVFSIQVTFFPNSGISIGVTNHHTLGDASTRFCFMKAWTSIARTGSDESFLANGTLPFFDRVVNYPKLDELYLKNVGVETIFSKGYQPPRLSGPTDKVRATFILTRTVINGLKKWVSTQLPTLPYVSSLTVLCAYTWSCIAKSRNDELEIFGIAVDCRARMDPPIPAAYFGNCVVLCLSIAKIDVLTGNEGFLNAAKLLGENLHKMLTDETGIVKDKWPFADSSSSQGIPTTIMGVAGTTKLKFYDMDYGWGKPTKYETISTDYSDSISLSTCKESNEDLELGVCLSATEMEAFVPIFSKGLEAYL >Ccrd_026143MEIEIISTENIKPSSPTPPHLKTFKLSLLDQLVSNPYVPLVLYYCNDNGDNVLQAHEKSLALKESLSKTLTQFYPLAGTIKDDLSIDXNDVGXYYASALVRCRLDEFLSHPDNNSTNTLLPFAPSFDVSGARARVTNVQVNVFECGGIAIXLCLSHKIVDGAXLXTFLKXWSNMXXGAXEVVHPNLTAPSLFPAKDLWXREASMAVCGSWLKEGKCSTKRFVFDXDXISTLKAEAARNGVQNPTRVEVXSALIWKSAMAASKXTCGFQKXSRLMHXVNLRKKLASTXSKXLIGNVLWIATADXQANXDXTLHGLAKNVREXVXKVDDEFVXKAQGDKGYIAMQQSVKEMGEIGSKGTMDNYXFTSWCRMGFYDIDFGWGKPGWVXGIVSHGSPVFMNLITLMDTKDGEGIEAWVNLDEEEMEILQCNSEPAPEFEAMEIEIISKESIKPSSPTPHHLKTFKLSLLDQLIINPYVPIVLYYPNHNGNNILQALERSLALKKSLSETLTQFYPLAGTIKNDLSIDCNDVGACYAIALVRCGLNELLSHPDHQLLNGLLPFQPSFEGSGAGARVTNVQVNIFECGGIAIGLCISHRIVDGAALRTFLDGWTNMACGAKEVVYPNLSAPSLFPAKDSWLRDSSMAMCGSWLKEGKCVTKRFVFDSDSIARLKAEATTNGVKSPTRVEVVSALIWKSAMEASKQTCGFQKPSRLAHLVNLRRKLASTLSKDSIGNVIWPATAKCQADYEPTLHGLVNKVRESISKIDSEFVTKAQGEKGYVAMQESIKEMGEISSRGTMDNYTFTSWCKMGFNQIDFGWGEPSWVTGIIGHGSPMFVNQVTLMDTKCGEGIEAFVNLDEEEMEIVQGNTKLQAYASVDPCPMPKIIDGAALCSFLKGWTNMACGAKEVVLKAEATRNGVKNPTRVAVVSALSL >Ccrd_003382MEELCVAKSSGSLVPPCAPTPPDTLHLSRIDRLPALRCNARTLHVFEALGPPGAAQSIILQALSKALVPYYPLAGRLISNPPQVQCSGEGVWFVEASANCTLQSVAYFEDVTSIPFDKLLPHHPPQTQAIDPLVLMQVTEFEGDGFVMGLTFCHTICDGLGAAQFLNAIAEFARGAHQLTISPVWHRDFLPQPQTITSCTPPPANFMLPPADYELEQANIDIPLHHINQLKQQFLASTFEIVAAILWRNRTKAISLGSSENRMMKLVFFANCRHLVQPPLPKGFYGNCFFPVTISAYSDTLSKAEMGEVVKMIQEAKANLGNEFADWVSIKKEEKEDPFAPPLGYGTLFVSEWGKLGFNQVDYGKGEAVHVVPMQGSSIIPVAIVGTMPRPNKGIRVMTWTISITSSFKPIPT >Ccrd_007714MAFKVEIKATHIVKPSSSTSDHLETHKLSLLDQLSPSVYPPIIFFYDTKQDSNLTPLLKASLSRVLASFYPYAGRINGDAFVDCNDDGIPYSEAIVDCSLSDVLKECDLNLMTQFVPLTEESVNPDHTIPLLVQVSFFKCGGIAIGACSSHKIGDAANFFTFIREWANVSLNDNLLLVPDFTISSLFPSIGSLNFNTGIKIPMNEKLVRKRFVFDASSISSLKAQTTPSTRVQAVSALIWKCAMNAVKTSVHGPDKPETKSSIAMTLVNMRGRLNPPLPETSFGNFVGSFLAEKRFDDDGEIELGGLVAQLRHGFKEFCDVYMKEVQDPKDGMLAILNYSKKIGEMLQRNGTEVFTFSSWCGFPLYEIDFGWGKPRWISVTNTPFRNGIMMMDTKEGNGIEVWANLEEDVMAIFEQDHHLDHIVLDHEIGVTTKRRRGK >Ccrd_002005MASDPILTVIEHSQISPPPVTVAPCSLPLTFFDLTWLLFPPVHHLFFYHFPHSKSHFIQTLLPNIKHSLSLALRHFFPFVSKLFVFNNSGVTRKPEIRHVEGDSVTLTVAECDLDFDDLTGDHPRKCEYFYPLIPPLGTAVKGSDYVTIPLFSIQITIFPGFGIAIGMTNHHSLGDANTRFGFLKAWATIACSGGDQSFLANGSLPIYERLIDIPKLDENKLRHTRVESFYQPPSLVGPTKVRATFVLTRTNIDRLKKRVLTHLPSLEYVSSFTVTCGYIWNCIAKSLVKMGEKKGDDELEQFILTVDCRSRLDPPIPVNYFGNCTAPCITTIQNVVLTGEKGFEIAAKLIGESINKMVNHKDGILKDAERWHEGFKIPARKIGVAGTPKLNFYDIDYGWGKPKKCETVSIDYNGSVSINACKNSTQDLEIGLCLSSMQMEAFANIFNDGIGNILS >Ccrd_017483MASLPILTVLEQCQVSPPPATVNDRSLPLTFFDILWLLFPPVHHLFFYDLPLTKTQFTETVVPSLKHSLSLTLQHFFPFVGNLIIFPTLNKKPEIRYVEGDSVAVTVAECNLDFDDLTGNHPRDCEKFYHLIPLLGQAAKVSDYITIPVFSIQLTFYPNRGISIGMTNHHSLGDASTRFCFLKAWTSIARSGTDESFLANGTFPLYDRLVNNPKLDESYLKYAKVETFNEEYRPQSLSGPTDKVRATFVLTRSTINLLKKQVSTDLPTLAYVSSFTVACGYIWSCIAKSRNDELELFGFAVDCRARLNPAIPAAYFGNCIGGCMAMAKTTLLTGKEGFVTAAKLLGENLHKLLTDKDGIVKDFSWYGDLFSNGVPTTMMGVAGTPKLKFYDMDFGWGKPRKHETVSIDYNNSISLSACKESNEDLEIGVCLSATDMEAFIPIFHDGLEAYR >Ccrd_009712MIEISNKTSSMVKPAERTWSGKLPLSELDQTGVTGHIPTFYFYTQSPHDWTTILQTLKSSLSSILVHFYPLAGRLSPVAGGRLELDCNAAGVQFVEAYADNKLTDLDTFLPLPIYHQLIPSIDYQNTPHEEIPLLVLQVTRFVCGGFCLSLSMSHTVADGEGALHFTCEWARISRGELLESPPYLDRKVLRAGDPPRASSSFEHAESNPPPILIEQSDNEPEHEKKTKVRMLKLTATQVEKLKKKANNSWKREMSRGFTRYEVITAHIWRTACNVRNHKPEQPTALAIAMNVRSKMRPPLPREYFGNAVIDAIATGCSGEIVSKPLGYSSSKIREAIERVDDEHVNSVIDFLKGQEDLSKFRELKSRSNGGGFCGNPNLGVTSWLTLPIYGADFGWGKEVHMTPGTHDNDGDSLILHGEDGDGSLVVALCLQVRHMEDFQKLVYNKTSCMVKPADQTWSGKLALSELDQTGVTVHVPTMYFYRQSPEDWFDVLQTLKSSLSSTLVHFYPLAGRLSSVSGGRLELDCNAAGVQFVEAYADKKLTDLDTFLPSPIYHQLTPSIDYVSAPLEEIPLLVLQVTRFVCGGLSLGLSISHVVADGQSALHFVSEWARISRGESLESPPYLDRKVLRAGDPPRANSRLEHTEFDPPPILIDHSENESEHEKETDVTMLNLTATQVEKLRNKANNSWKSEMSHGFTRYEAITAHIWRTACKVRNHKPEQPTALAICIDVRGKMSPPLPRKYFGNAIINVIATGRSGEIVSKPLGYSSSKIRDAIEKVDDEHVNSVIDFLKGQEDLSKFRDLQPSSNDGHFYGNPNLGVISWLTLPLYGADFGWGKEIHMGPGTHDSDGDSLILHGKDGDGSLVVALCLQVRHMEDFQKVFYQDIDIE >Ccrd_020278MKNYYHLLTLRRSKGLFSSNSRWVHQTSTAIQRQHGHRHPPPPPPLPPPSSHPHISRNTCRPINAFVSHHCLQTTRSNLTSNHYYHSQSSTLHTSQLQLPYHEDDSSPLHYHVTVKGRDVISSAKSNQRYWLPLSNLDLLLPPVEAGVFFCYRKKDRDMSSESVVNSIKRSLAGILSSFYPLAGEIVANKQGEAEVVCNNGGVEFVHAHADIELKDLDLHHPDDSVKGKLVTEFNCGAIIISCAIDHRVADAYSLNMFLVAWAKYAKSGTMSDVDIPSFRPSIFNSRRPPTYAKSLDNLYIPISSIPPPSSFDQGPLHSRMYYIHAQSIDHLQSEASSQESKRSKLLSFTAFLWKLLAHGGDDAVNTTSRMGVVVDGRRFLADEQPSSPEKNHFGNVLSVPFGVATHSDLKAMPLNEIADRVHRFVAEATNEEHFRGLIDWVELHRPKPAVARIYFGVEKSEGEAVVVSSGRDLPINDMDFGWGRPEFGSLHFPWGSRTGYISTMPSGKRIGDWVVYVHLKQKDLDLIERMAPNVFKPLTHSLAFD >Ccrd_015561_HCT_DQ104740MKIEVRESTMVRPAEETPRINLWNSNVDLVVPNFHTPSVYFYRPNGAANFFDPKVMKDALSRALVPFYPMGGRLKRDEDGRIEIDCQGQGVLFVEAESDGVIDDFGDFAPTLELRKLIPAVDYTLGIESYSLLVLQVTYFKCGGVSLGVGMQHHAADGASGLHFINTWSDLARGLDLTVPPFIDRTLLRARDPPQPAFDHIEYQPAPPMKTAPTPTPTDDESVPETTVSIFKLTRDQVNALKGKSKEDGNTINYSSYEMLSGHVWRCVCKARGLPDDQDTKLYIATDGRARLRPSLPRGYFGNVIFTTTPIAVAGDLQSKPTWYAASKIHDALARMDDDYLKSALDYLELQPDLKALVRGAHTFKCPNLGITSWARLPIHDADFGWGRPIFMGPGGIAYEGLSFVLPSPINDGSLSIVISLQAEHMKLFSNFLYDI >Ccrd_013366MVSSKEESLIHDIKISTVGPGYVSGQGAVQELTSMDLAMKLHYLRTTYYFRSQALEGLTIINIKETMFYWLNHCYIPCGRFRRSESGRPYIKCNDCGVRFIEARCTITLDEWLELRDDARHKLLVPNQVLGPDLSFCPPLTKFKCGGTSIGVSWAHVLGDAFSAAGFMNLWGQATKRQYPTQPLRMTRSDNMGHNPKSPIKDPLAVKRVGPVGDHWTSSNHSKMETYSFSVSWPELTRLQSKICGDKNHQQMPPFETICTVVWQCVAKAKHGSEVKVVTICKHGSKKIFEGVITNEAQFIKVVKTESSVEESSLMELGLLIMNQGVDVRRNVKEAMETNTELPDFLVYGANLTFVDLYDVPFYELDVRGQTPVYVNCAIDNIGDEGVVLVLPARKNHSDGMTVSITLPTDHISKFRSVLKKEWSL >Os_cl_1a_LOC_Os06g05320.1MSSLVRVLAVSHVLPDEVAAGGAWPPPPPHVVELSFLDNLQVSKAAIQRLFFYDGGSLPPFESVDRSLQSSLAAVLAIFLPLAGKLAYLPEPGDVVIDYSPDAVSPGVKFVEAEYSGSVDDMRRLASDDEHHTEAFLQLVPELEVSMLPAPLLAVQVTRPRDDHAGGGGGAVAVGVAIHHGVADGQSVWQFIKAWAAAARGGSPAGQGLVPPTFDRSRIRHPTADSHELAHTILHKMSPALPMVTPRSKPADMAQQRRRTFLLSAGEIQSLKQRISESETGGELLHNRLSTYVTISSLAWTSIVRAKCGALDAAADDVYFMVSADCRRRLRPPADEGYFGNCIAIAIARASAGELLDDDGLAGLARAAAAIQAAIRDELELEDPVGGAERWAERLAAIPRGRLTAAGSSHRFMAYETDFGWGAPSRVELVTVYGNELVAMLGGAADGGVQVSVVLGRALMDAFADNFRRQVVACPNSTVSRSRHH >Os_cl_1a_LOC_Os02g57480.1MATKVVDKLTVAASPPADGGVLPLTFFDVPWIFTGPVERVFLYTYPHAVEHLAAHLLPSLASSLSAALHRFYPLLGRVRPCSSGGGGGGYEFCSTGGDADRVELTVAESGDDFEELAGGGPMDVGRLYSLVPRLPRPEEGSSELAAVQVTVFPGKGLAVGVSIHHVACDDSSFMHFVKTWAANCRVASGGDVDAVPPPPPPFLDRGVVADPDGLAAKTLDQMRQLANSGPPPPPPSGPPPKLFMASFTLTRDSIDKLKQRVTASGGGGVHCSAFTVACAYAWTCLARVDATSAARERAHLLFSVECRRRLTPPVPQEYLGNCLRPCFVEVDTAGLLGSGADGVVTAAVAIGAAIRGLDDGVLDGADGWFQKIVSLMPHRPMSIGGSPRYGVYDTDFGLGRPAKVELLSIDKTPGTVSMAEARDGHGGIEIGVALPEADMARFSSCFADGLKQL >Mt_cl_1a_Mat3MASLNKHIKIHEQCKVSPSSSSTQLSLPLTFFDYIWLRFHPVERIFFYTLPSSHSHPTFFFENLVPKLKSSLSLTLQHFLPLAGNIVWPSDSPKPFLQFNPNDDGVSLLLAQCDDDDVSFDKILEHNSPQEASLSRSFVPHLESSDSFASIISIQITLFPKNGFSIGISTHHAVLDGKSSTMFIKAWSSICKSLEEETQSLNLEPLLEPFLERELIEDPNDFENSFINTWNRISSHFDKSSVKSIKIMSSMFQPIIKDAVRETFELTREDLEKINKRVFSKWNNIEDGAQEKEQEQPKKLSTFVLTCAYVSVCIAKAIQQSESDKKQKFSIGFPVDCRSRLVPPIPKNYCGNCVSNHIVDTEPYDFTKEDGVVIVAKKIYGKTQEMDKGFLDGIETMMYKYMAMIGEGVKGIGVAGSTRFGVYEIDFGFGRPAKVEITSIDRGLTIGLTESKDLKGGVEIGLVLEKHVMDLFQAIFREGLCFD >At_cl_1a­_MaT3MVNFNSAVNILEVVQVSPPSSNSLTLPLTYFDLGWLKLHPVDRVLFYHVPELTRSSLISKLKSSLSATLLHYLPLAGRLVWDSIKTKPSIVYSPDDKDAVYLTVAESNGDLSHLSGDEPRPATEFHSLVPELPVSDESARVLAVQVTFFPNQGFSLGVTAHHAVLDGKTTAMFLKAWAHNCKQEQEALPHDLVPSLDRIIVQDPTGLETKLLNRWISASNNKPSLKLFPSKIIGSDILRVTYRLTREDIKKLRERVETESHAKQLRLSTFVITYAYVITCMVKMRGGDPTRFVCVGFASDFRSRLNPPLPPTFFGNCIVGSGDFDVKAEPILEEGEGKGFITAVETLTGWVNGLCPENIEKNMLLPFEAFKRMEPGRQMISVAGSTRLGIYGSDFGWGKPVKVEIVTIDKDASVSLSESGDGSGGVEVGVCLKKDDVERFGSLFSIGLE >Pt­_cl_1a­_POPTR_0004s09280.1MNILEVCHVPPFSSSFKSTPTEFSLPLNFSDIFNLKFPPVESIFFYKLGESTNTFFKTEILPKIKHSLSITLFHFLPLAGYLSWPQNSKKPIITYNTADDGVLLTVAESNEDFDHLYSEVRYASESHPYLAPLFVSDTKASILSFQITLFPNKGFTISYTLNHAVLDGRSISLFMNSWAYICRNLDENVKISPSSLPEELIPSFDRTVIPGSEGLEMRYLNYWLGLKLPGSDANPRSLEPIPFPVPADVVRATFGFSREDIKKLGERVLSKLENGNQSKPFSTYVLAYAYTLVCMVKAKGLKNNNKVKFGLTTDCRPRLNPPLSRNYIGNCVTSCDVLVEAEHLLKETGVVYAAKRLNEMIEGLENGVLELAKGRVPFMDVEPGVRIILVAGTNRFGKYGADFGWGKPTNVEITTIDVGESLSMMESRDESGGVEIGLVLKKHEMEIFDSLFVHGLKVLRSSL >Nt­_cl_1a­_BAD93691.1MASVIEQCQVVPSPGSATELTLPLTYFDHVWLAFHRMRRILFYKLPISRPDFVQTIIPTLKDSLSLTLKYYLPLAGNVACPQDWSGYPELRYVTGNSVSVIFSESDMDFNYLIGYHPRNTKDFYHFVPQLAEPKDAPGVQLAPVLAIQVTLFPNHGISIGFTNHHVAGDGATIVKFVRAWALLNKFGGDEQFLANEFIPFYDRSVIKDPNGVGMSIWNEMKKYKHMMKMSDVVTPPDKVRGTFIITRHDIGKLKNLVLTRRPKLTHVTSFTVTCAYVWTCIIKSEAATGEEIDENGMEFFGCAADCRAQFNPPLPPSYFGNALVGYVARTRQVDLAGKEGFTIAVELIGEAIRKRMKDEEWILSGSWFKEYDKVDAKRSLSVAGSPKLDLYAADFGWGRPEKLEFVSIDNDDGISMSLSKSKDSDGDLEIGLSLSKTRMNAFAAMFTHGISFL >At­_cl_1a­_At3AT2MAAQLQPYNIIETCHISPPKGTVASTTLPLTFFDAPWLSLPLADSLFFFSYQNSTESFLQDFVPNLKHSLSITLQHFFPYAGKLIIPPRPDPPYLHYNAGEDSLVFTVAESTETDFDQLKSDSPKDISVLHGVLPKLPPPHVSPEGIQMRPIMAMQVTIFPGAGICIGNSATHVVADGVTFSHFMKYWMSLTKSSGKDPATVLLPSLPIHSCRNIIKDPGEVAAGHLERFWSQNSAKHSSHVTPENMVRATFTLSRKQIDNLKSWVTEQSENQSPVSTFVVTLAFIWVSLIKTLVQDSETEAKDEVFHLMINVDCRNRLKYTQPIPQTYFGNCMAPGIVSVKKHDLLGEKCVMAASDAITARIKDMLSSDLLKTAPRWGQGVRKWVMSHYPTSIAGAPKLGLYDMDFGLGKPCKMEIVHIETGGSIAFSESRDGSNGVEIGIALEKKKMDVFDSLLQKGIKKFAT >Os_cl_1b_LOC_Os05g37660.1MVEAASAAPATVVVPLTQTLCANAPATSVTVVSKQTVRPDGASAVGDVKLSVSDMPMLSCHYIQKGLFFPPPGVPIASLVSSLVCALSRALAVFPALAGRLVTLDDGRIVIRCDGVAVEFYHAVAPALSLGDFLVPNADVPTRLTKDLFPMDRTVSYDGHRRPLTSFQLTVLGDGAVFVGFVANHAVVDGTSFWHFFNTWAGLCRGTPVQPPDLRRNFFGDSTAVIRFPGGAGPAVTFDADAPLRERVLHFSAAAIRELKAKANQWKRSDKFAEANGKHVDETKAHGGYREISSFQSLCAHIWRAVTRSRRLLAADATTTFRMAVNCRHRLRPAISPLYFGNAIQSVATTATVAELASNDLRWAAARLNATVVAHEDGAIRRAAAEWEAAPRCFPLGNPDGAALTMGSSPRFPMYDGNDFGWGRAIAVRSGRANKFDGKMSAFPSQAGDGSVDVEFCLAPDTMARLLGDHEFLQYVSRAP >At­_cl_1b­_AT5G38130.1MPFDLFLSYKVFHLLFYRRKMVEVTVISSTMVRPENINQTGRQKIHLTPHDLDLLYLFYPQRGLLFHKPDPENSIIPRLMASLSTALEIYFPFAGRLVKVNNHEDDTVSFYIDCDGLGAKFVHAKAESITVNDVLQSHGSVPYFISKFFPANNVQSRDALVSEPLLALQVTEMKDGVFISFGYNHMVADGTCFWKFFHTWSKICLNGSDPSIQSIVLKDWFCDGIDYPVHVPVLEMETLPRWEPSTKERVFHLSKKNILDLKAKANNEIDTNDLKISSLQAVVAYLWLSIIRHSGLNREEETQCNVAADMRPRLNPLLKKECFGNVTNLATATTTVGELLDHGLGWTALQISKSVRSETNESYEVFAKNWVRNVKRPKTSFGSRLANNSLIISSSPRFEVYEHDFGWGKPIAARAGPADGAGGMLVMFRGVEEGSIDVHATLNSSLWSDVLVNLLTNDMVGQ >Mt­_cl_1b_AC122728_6MMSTHCLKLVSECFIKPYPPIEDSKQICYLAPWDIVMLSANYIQKALLFKKPTSSLNQQHFIDKLKHSLSLTLFHFYPLSGRLVTKKSEDPHSYTVFVDCKNSLGAKFIHATLDITINDILSPIDVPPIVHSFFDHHRAVNHDGHTMSLLSIQVTELLDGVFIGCSMNHAVGDGTAYWNFFNTLSEIFQKVVDVDVHIPVPISHQPIHNRWFPEGYGPIINLPFKHHDEFIHRYEAPILRERIFHFSAEFIAKLKAKANKECETTKISSFQSLSALVWRSITRVRRLHHDQRTTCKLAINNRTRMEPSLPKEYFGNSVYAVSTETTKGELLENGLGLASWKIHLAIAKYDHRVVRQLVEEWLRSPIVVRMDMLVDPYSVMMGSSPRFNMYGSEFGMGKALGVRSGYANKFDGKVTSYPGQEGGGSVDLEVCLSPEKMTLLETDEEFMNSVSVFNPLF >At­_cl_1b­_At5g23940MKIKIMSKTHVKPTKPVLGKKQFHLTTFDLPYLAFYYNQKFLLYKFQNLLDLEEPTFQNEVVENLKDGLGLVLEDFYQLAGKLAKDDEGVFRVEYDAEDSEINGVEFSVAHAADVTVDDLTAEDGTAKFKELVPYNGILNLEGLSRPLLAVQVTKLKDGLAMGLAFNHAVLDGTSTWHFMSSWAEICRGAQSISTQPFLDRSKARDTRVKLDLTAPKDPNETSNGEDAANPTVEPPQLVEKIFRFSDFAVHTIKSRANSVIPSDSSKPFSTFQSLTSHIWRHVTLARGLKPEDITIFTVFADCRRRVDPPMPEEYFGNLIQAIFTGTAAGLLAAHGPEFGASVIQKAIAAHDASVIDARNDEWEKSPKIFQFKDAGVNCVAVGSSPRFRVYEVDFGFGKPETVRSGSNNRFNGMMYLYQGKAGGISIDVEITLEASVMEKLVKSKEFLLSEEEEEDDGKKLTNGNGHVNGNGNGYVNGNGNGFV >Os_cl_2_LOC_Os03g53360.1MPTAAAMAGGMATGPGSRVTRYAKSTAASVTPVRPGKTHALSALDNAMERHAVYLVLYYRAAPGLDREPLKESLSDVLSQYPAMTGRLTRPAAAAAGGGGEGGEGGGATAAVHHGWIVKCNDAGVRTVDATAAATLDEWLATASGEEEMDLAYFEPMGPDPYIWSPFYVQLTEFADKSYALGLSCTHLHNDPTAAVLFLNAWAAAHRRDSPYPPFLHSPALAAKSAAPPPEHPLLAAKSRGSPDTGGEMSSATFRFSAAAMRALLSAVEPGTTPFAALAALFWLRVAAAAADAAAGGGAAEERELTLALDFRKRMQAPLPTGYYGTAVHFATARADLSSGLASVAAAVERRAAAVPEEELWPAIEWLHTRQAAGGEPFQMYGPELTCMALDHVPLYGAEFAAGAAPARAACRVGGASGEGLVIVLPSAEGESARDVAVTLPAAVTARICRDGEVLRYGADVVFGPKVDTQAS >Cr_cl_3a_AAC99311.1MESGKISVETETLSKTLIKPSSPTPQSLSRYNLSYNDQNIYQTCVSVGFFYENPDGIEISTIREQLQNSLSKTLVSYYPFAGKVVKNDYIHCNDDGIEFVEVRIRCRMNDILKYELRSYARDLVLPKRVTVGSEDTTAIVQLSHFDCGGLAVAFGISHKVADGGTIASFMKDWAASACYLSSSHHVPTPLLVSDSIFPRQDNIICEQFPTSKNCVEKTFIFPPEAIEKLKSKAVEFGIEKPTRVEVLTAFLSRCATVAGKSAAKNNNCGQSLPFPVLQAINLRPILELPQNSVGNLVSIYFSRTIKENDYLNEKEYTKLVINELRKEKQKIKNLSREKLTYVAQMEEFVKSLKEFDISNFLDIDAYLSDSWCRFPFYDVDFGWGKPIWVCLFQPYIKNCVVMMDYPFGDDYGIEAIVSFEQEKMSAFEKNEQLLQFVSN >Fa_cl_3a_AAG13130.1MEKIEVSINSKHTIKPSTSSTPLQPYKLTLLDQLTPPAYVPIVFFYPITDHDFNLPQTLADLRQALSETLTLYYPLSGRVKNNLYIDDFEEGVPYLEARVNCDMTDFLRLRKIECLNEFVPIKPFSMEAISDERYPLLGVQVNVFDSGIAIGVSVSHKLIDGGTADCFLKSWGAVFRGCRENIIHPSLSEAALLFPPRDDLPEKYVDQMEALWFAGKKVATRRFVFGVKAISSIQDEAKSESVPKPSRVHAVTGFLWKHLIAASRALTSGTTSTRLSIAAQAVNLRTRMNMETVLDNATGNLFWWAQAILELSHTTPEISDLKLCDLVNLLNGSVKQCNGDYFETFKGKEGYGRMCEYLDFQRTMSSMEPAPDIYLFSSWTNFFNPLDFGWGRTSWIGVAGKIESASCKFIILVPTQCGSGIEAWVNLEEEKMAMLEQDPHFLALASPKTLI >Cb_cl_3a_AAC18062.1MNVTMHSKKLLKPSIPTPNHLQKLNLSLLDQIQIPFYVGLIFHYETLSDNSDITLSKLESSLSETLTLYYHVAGRYNGTDCVIECNDQGIGYVETAFDVELHQFLLGEESNNLDLLVGLSGFLSETETPPLAAIQLNMFKCGGLVIGAQFNHIIGDMFTMSTFMNSWAKACRVGIKEVAHPTFGLAPLMPSAKVLNIPPPPSFEGVKFVSKRFVFNENAITRLRKEATEEDGDGDDDQKKKRPSRVDLVTAFLSKSLIEMDCAKKEQTKSRPSLMVHMMNLRKRTKLALENDVSGNFFIVVNAESKITVAPKITDLTESLGSACGEIISEVAKVDDAEVVSSMVLNSVREFYYEWGKGEKNVFLYTSWCRFPLYEVDFGWGIPSLVDTTAVPFGLIVLMDEAPAGDGIAVRACLSEHDMIQFQQHHQLLSYVS >Ps_cl_3a_AAK73661.1MATMYSAAVEVISKETIKPTTPTPSQLKNFNLSLLDQCFPLYYYVPIILFYPATAANSTGSSNHHDDLDLLKSSLSKTLVHFYPMAGRMIDNILVDCHDQGINFYKVKIRGKMCEFMSQPDVPLSQLLPSEVVSASVPKEALVIVQVNMFDCGGTAICSSVSHKIADAATMSTFIRSWASTTKTSRSGGSTAAVTDQKLIPSFDSASLFPPSERLTSPSGMSEIPFSSTPEDTEDDKTVSKRFVFDFAKITSVREKLQVLMHDNYKSRRQTRVEVVTSLIWKSVMKSTPAGFLPVVHHAVNLRKKMDPPLQDVSFGNLSVTVSAFLPATTTTTTNAVNKTINSTSSESQVVLHELHDFIAQMRSEIDKVKGDKGSLEKVIQNFASGHDASIKKINDVEVINFWISSWCRMGLYEIDFGWGKPIWVTVDPNIKPNKNCFFMNDTKCGEGIEVWASFLEDDMAKFELHLSEILELI >At­_cl_3b­_At5g16410MIHFYSSSLHSSKFLDGFRYHSLSVIRSEARSFNVTTTRKEVVVAAAPLLSLPENRVIPLSNLDLLIPPVDINVCFFYKKPLYGIIGDALKTAMAEALVSYYVLSGEVSINPTNGENEILCSNGGVEFVEAAADVELRELNLYEPYQSIAKFVPMKKHGVFAIQVTELKCGSVVVGCTFDHRIADAYSMNMFLVSWAEISRSDIPISYVPLLKRSLLKPRRPLIIDSSIDKLYMPITSLTVPQEITNQDNILTSRIYYIKADVLEKFQTLATNGKRTKLESFSAFLWKLLAKHAATESVLPTKTSKLGIVVDGRKKLMEQENCNYFGNVLSVPFGERRIDDLIHKPLSWVTDEVHKLLESTMTKDHFLNLIDWVETSRPIPVISRIYSTGSNDGPAFVVSSGKSFPVARIDFGWGSPVFGSYHLPPGSRAGYVMTMPSPVENGGSGDWTVYLHLTKGQLRFIEQEASHVFNPVDNDYLKI >Vv_cl_3b_GSVIVP00006814001MGGGDFTVTVSRKEVVAAVLPVQEYWLPLSNLDLLLPPVDVGVFFCYKKPHDLTFGSMIGVLKEALAQALVSYYPFGGEVLSNSAGEPELLCNNRGVDFMEAYADVQLQNLNLYNPDESIESKLVPKKKHGVLSVQVTELKCGGVVVACTFDHRIADAYSANMFLVSWAEMAQSKPLSVLPSFRRSLLNARRPGSYDPSLDDMYVPMSALPPPKAPQSGDDLLINRLYYVTAEQLSLLQSLATSKSSSCKRTKLESLSAFLWKMVAKSAVTENANQKICRMGTVVDGRKRLSSGDEVKAAMMASYFGNVLSIPFGKKTINELKEKPLSWVADAIHEYLEGAVTKEHFLGLIDWVEAHRPEPALAKIYSSGSRDGPAFVVSSGQRFPASRVDFGWGMPALGSYHFPWGGEAGYVMPMPSPVRDGDWVVYMHLSVGQVEWIETEAAHIFRPLSSEYLNLSNSD >Os_cl_3b_LOC_Os11g42480.1MAPEAAVPAGEDLTIRVVSRRLVKASDATIQPHVATVSNLDLYFNNYQASMVCLYPSNLPVAGVAGSFDAVVAAFEAGLPSLLNHFYPLAGRIVVDPVSRLPELHCHNQGAELVVGEVDAALGSLDFAGMDGSLRRILLPYPDDVMLSVQLLRFACGGFSVVWGNNHLPNDGHGISMVVRMCPPSYGAAVRATFAAYHDESRLVNVLTTQDSFVERLYYIEAGDVARLRDMASTGQRRASRVQAVSAFLWKALAGVVAASRVPEERCRMGWWVDARRRVASPALVPAMHSFFGNMTAYALGEAAVEEILERPLAEVAAMAREAIASIAYDAYVQELVDWVEEHKAEKMMEVSALGLGSPTVNQTVFASFPLDTDFGFGEATLAMPVWENGRVSSGTLAVGARPGGDGSWLVSAYIWPRLAAALESDDHRIFKPLTAAYLGFV >At­_cl_3b_At1g31490MKIFDVSFTGEFIVKASGDQPEKVNSLNLSNFDLLSGRFPVTYFYFYPKQPQLSFETIVKSLQSSLSQTLSYFYPFAGQIVPNETSQEEPMIICNNNGALFVEARAHVDLKSLDFYNLDAFLQSKLVQVNPDFSLQIQATEFECGGLALTFTFDHALGDASSFGKFLTLWSEISRNKPVSCVPDHRRNLLRARSPPRYDPHLDKTFIKCSEEDIKNIPMSKTLIKRLYHIGASSLDALQALATVNGESRTKIEAFSAYVWKKMVDSIESGHKTCKMGWLVDGRGRLETVTSSYIGNVLSIAVGEASIENLKKNHVSDIANIVHKSITEVTNDTHFTDLIDWIESHRPGLMLARVVLGQEGPALVLSSGRRFPVAELDFGFGAPFLGTVCSTVEKIGVGYLNQRPSACNDGSWSVSAIVWPELATALESDSVFQPMSAKHLQLQT >Hv_cl_4_AAO73071.1MKITVHSSKAVKPEYGACGLAPGCTADVVPLTVLDKANFDTYISVIYAFHAPAPPNAVLEAGLGRALVDYREWAGRLGVDASGGRAILLNDAGARFVEATADVALDSVMPLKPTSEVLSLHPSGDDGPEELMLIQVTRFACGSLVVGFTTQHIVSDGRSTGNFFVAWSQATRGAAIDPVPVHDRASFFHPREPLHVEYEHRGVEFKPYEKAHDVVCGADGDEDEVVVNKVHFSREFISKLKAQASAGAPRPCSTLQCVVAHLWRSMTMARGLDGGETTSVAIAVDGRARMSPQVPDGYTGNVILWARPTTTAGELVTRPVKHAVELISREVARINDGYFKSFIDFANSGAVEKERLVATADAADMVLSPNIEVDSWLLIPFYDMDFGGGRPFFFMPSYLPVEGLLILLPSFLGDGSVDAYVPLFSRDMNTFKNCCYSLD >Vv_cl_5a_GSVIVP00029327001METNFVRVKEAVLVAPSETTPSRVLALSALDSQLFLRFSIEYLLVYQSRPCSDHGVTADHVKAALGRALVPYYPLAGRVRVGSSGSNLEVVCRAQGAVFIEAVSDLTVADFERTPRHVTQWRKLLSLYVEDVLRGAPPLVVQLTWLADGGAALGVGINHCLCDGIGSAEFLNAFAELATGRGGLSEFKPKPVWDRHLLDPKPLEPPRRASSSTHPEFNSVPDLSGFVTRFSQEPLVPTSITFDKRCLNELKQLASCTGRPSESSYTSFEILSAHVWRSWARALNMPSNQTLKLLFSINIRNRVKPSLPPGYYGNAFVLGCAQTTVKEITEKGLGHMCGLVKRAKERVGDEYVRRVVDMVSGESRASPDSVGVLILSQWSRLGLERVDFGMGIPVNVGPICSDRYCLMLPVSDQRDAVKVMVAVPTSAVDQYIHLIGNSGVGGSGWRRSGSLASARVNGSAPSPPLRTPLEPTTVQPSFSTAAELSSTSNRPSPPPRPPAAEARRRRAALKRSAHCSLVHLGSASLSLLPLPPRTRRGDTCRTGSATVWIRPYYVETWCRVNSNWCNSSISCYIHRLI >Tc_cl_5a_AAL92459.1MKKTGSFAEFHVNMIERVMVRPCLPSPKTILPLSAIDNMARAFSNVLLVYAANMDRVSADPAKVIREALSKVLVYYYPFAGRLRNKENGELEVECTGQGVLFLEAMADSDLSVLTDLDNYNPSFQQLIFSLPQDTDIEDLHLLIVQVTRFTCGGFVVGANVYGSACDAKGFGQFLQSMAEMARGEVKPSIEPIWNRELVKLEHCMPFRMSHLQIIHAPVIEEKFVQTSLVINFEIINHIRRRIMEERKESLSSFEIVAALVWLAKIKAFQIPHSENVKLLFAMDLRRSFNPPLPHGYYGNAFGIACAMDNVHDLLSGSLLRTIMIIKKSKFSLHKELNSKTVMSSSVVDVNTKFEDVVSISDWRHSIYYEVDFGWGDAMNVSTMLQQQEHEKSLPTYFSFLQSTKNMPDGIKMLMFMPPSKLKKFKIEIEAMIKKYVTKVCPSKL >Ms_cl_5a_CAC09063.1MSFAVTRTSRSLVTPCGVTPTGSLGLSAIDRVPGLRHMVRSLHVFRQGREPARIIREALSKALVKYYPFAGRFVDDPEGGGEVRVACTGEGAWFVEAKADCSLEDVKYLDLPLMIPEDALLPKPCPGLNPLDLPLMLQVTEFVGGGFVVGLISVHTIADGLGVVQFINAVAEIARGLPKPTVEPAWSREVIPNPPKLPPGGPPVFPSFKLLHATVDLSPDHIDHVKSRHLELTGQRCSTFDVAIANLWQSRTRAINLDPGVDVHVCFFANTRHLLRQVVLLPPEDGYYGNCFYPVTATAPSGRIASAELIDVVSIIRDAKSRLPGEFAKWAAGDFKDDPYELSFTYNSLFVSDWTRLGFLDVDYGWGKPLHVIPFAYLDIMAVGIIGAPPAPQKGTRVMAQCVEKEHMQAFLEEMKGFA >Os_cl_5a_LOC_Os01g24790.1MAMELSFTVHRREAVLVGPSVSTPCETKRLSDIDDQESLRYHVPGLLVYRGGQPPAPCVRDNDPSGIIRAALSRALVHYYPLAGRLREVEGRKLVIDCSGEGVVFVEADADVRLEEMEAAAAGGHGLRPSFPCVDQLVPDVRSSGRGGSVLSCPLVGIQVTRLLCGGFIVGTAVNHSVCDAMGIVQFLNAVADIAGGLPAPAVHATWSRELLDARSPPAPAFPHREYDMIDILPGGGREADNIVRSFLFSSTDIAALKEEALLPHPEHRLRGGSSTATSFEVLAAFLWRARTAALEVPADEEVRLVAVVGFSRIAALGLPSGYYGNTCAYPTVVMTAGELLRGCTLGDVVRLVQEAKAAVTAEYVRSTAECLVLRRRPRLARTNLFVVTDVRRVGFDRVDFGWGDPVYGGPARALPTVSLLVNVKGSSNVVGAVVSLPSLVMGRFSAELESFLNT >Vl_cl_5a_AAW22989.1MASPSSPLVFSVNRCVPQIVRPANPTPREVKQLSDIDDQEGRRFQIPVIMFYRNNPLMEGKDPVKVIREALGKALVYYYPFAGRLIEGDNRKLMVDCTGEGVLFIEADADTTLENLGDAIQPMCPCFEELLYDVPGSTTILGSPLILIQVTRLRCGGFIFALRLNHTMSDAAGLIQFLDTIGEMAQGLSVPSLLPIWQRELLNARNPPRITRIHHEYEKVTNTKGTLMAMDENSLVHRSFFFGREEIRALRNRLPASLGACSTFEVLMACVWRCRTIAFAVDPDEVVRISCIINMRGKHGFELPPGYYGNAFVTPASITKAGMLCKNPLEFAIRLVKKAKAEMSQEYIKSVADLMVIKGRPLFTQPGNFTVSDVTRAGLGEVDFGWGKPVYGGVARACPIISFRMLFRNSKGEEGSVIPIWLPPPVMERFEQELKRMTKKAELLITSML >Nt_cl_5a_AAN09798.1MDSKQSSELVFTVRRQKPELIAPAKPTPREIKFLSDIDDQEGLRFQIPVIQFYHKDSSMGRKDPVKVIKKAIAETLVFYYPFAGRLREGNGRKLMVDCTGEGIMFVEADADVTLEQFGDELQPPFPCLEELLYDVPDSAGVLNCPLLLIQVTRLRCGGFIFALRLNHTMSDAPGLVQFMTAVGEMARGASAPSILPVWCRELLNARNPPQVTCTHHEYDEVRDTKGTIIPLDDMVHKSFFFGPSEVSALRRFVPHHLRKCSTFELLTAVLWRCRTMSLKPDPEEEVRALCIVNARSRFNPPLPTGYYGNAFAFPVAVTTAAKLSKNPLGYALELVKKTKSDVTEEYMKSVADLMVLKGRPHFTVVRTFLVSDVTRGGFGEVDFGWGKAVYGGPAKGGVGAIPGVASFYIPFKNKKGENGIVVPICLPGFAMETFVKELDGMLKVDAPLDNSNYAIIRPAL >Os_cl_5a_LOC_Os05g22300.1MAGAPTLAFSVRRRERELVAPAKPTPYEFKMLSDIDDQDILRFNRSGILFYRHSPSKDGLDPVKVIKAAISETLVHFYPVAGRFRELRPTRKLVVECTGEGVVFVEADANFRMDELGTSLAPPVPCYDMLLCEPESPTADVVDRPLLFIQVTRLACGGFVFGMHICHCMADGSGIVQFLTALTEFARGVHGAPTVRPVWEREVLTARWPPTVTRDHVEYTPLPNPGKDVLSPTDAYAHHVFFFGASEIAALRSQAPPDLRAVSSRFDLVGAFMWRCRTAALRYDPGDVVRLHMFVNARVRNRSKRPVPRGYYGNAIVFAAASVPAGELWRRPFGYALRLLMQAKARASEEGYVQSVANFNAAHRRPPFPKARTYLISDMTQAGLMAIDFGWGKPVYGGPATTMLATFHLEGRNEVGEAGVIVPIRLPNPVIERLIQEVNKGLTAGAVADAKANVVPDDCVLAKL >At­_cl_5b_At2g19070MAPITFRKSYTIVPAEPTWSGRFPLAEWDQVGTITHIPTLYFYDKPSESFQGNVVEILKTSLSRVLVHFYPMAGRLRWLPRGRFELNCNAEGVEFIEAESEGKLSDFKDFSPTPEFENLMPQVNYKNPIETIPLFLAQVTKFKCGGISLSVNVSHAIVDGQSALHLISEWGRLARGEPLETVPFLDRKILWAGEPLPPFVSPPKFDHKEFDQPPFLIGETDNVEERKKKTIVVMLPLSTSQLQKLRSKANGSKHSDPAKGFTRYETVTGHVWRCACKARGHSPEQPTALGICIDTRSRMEPPLPRGYFGNATLDVVAASTSGELISNELGFAASLISKAIKNVTNEYVMIGIEYLKNQKDLKKFQDLHALGSTEGPFYGNPNLGVVSWLTLPMYGLDFGWGKEFYTGPGTHDFDGDSLILPDQNEDGSVILATCLQVAHMEAFKKHFYEDI >cl_4_LOC_Os10g23820.1MAVAVEITRSEVLRPSETLAAGGGGKRSQLTVFDRAAMDWYIPAVFAWDGAAAPSNDEVKGGLAAVLARYPHLAGRFDVDERGRRCFNLNNAGVRVLEATVAADLADALAHDVAAHVNELYPKADMENADEPVFQVQLTRYACGGLVIGTACNHQVSDGQSMSFFYVAWAAAVRSAGATLPTPFVDRAAIAVPRGPPAPAFDHRNIEFKGEHSWTHSYGSLPLERIRNLAVHFPDEFVAGLKSHVGARCSTFQCLLAHAWKKITAARDLSPEEYTQVRVAVNCRGRASPAVPMDYFGNMVLWAFPRMRVRDLLSSSYAAVVGVIRNAVARVDEQYIQSFVDFGEVAAGDELTPTAAPPGTVFCPDLEVDSWLGFRFHDLDFGRGPPCAFLPPDVPVEGLLIFVPSCAAKGGVEMFMALDDVHVEAFRQICYSMD >cl_4_LOC_Os09g37200.1MEVKVLSSRLVRPSYPASAAAPEEEFVPSSMFDKVTYDMQMAIIYAFRPPGPSVADIEKGLAAVLGVYRLFAGQVVRGGGGELRGVVLNDHGARLVEACVDGSLADIAPAKPSPVVLRLHPSLEGEIEEVVQVQLTRFACGSLAVGFTANHAVADGHATSDFLVAWGRAARGLAVAATAAAPPHHHPGMFRPRDPPLVEFEHRGVEYYRPPPPAAGVDGDVGGDHKQQHGHGGEEASHGIVIHKAHFTKDFIARLRAAASEGRGRPFSRFETILAHVWRTMTRARGLGNPLQSSTIRISVDGRQRLSAPAGYFGNLVLWAFPRATVGDLLGRPLKHAAQVIHDAVARADAAYFRSFVDFASSGAVEGEGLAPTAVLKDVLCPDLEVDSWLTFPFYELDFGGGCPTYFMPSYFPTEGMLFLVPSYLGDGSVDAFVPVFDHNLEAFKQSCYSIE >Nt_cl_5b_CAD47830.1MKIEVKESTMVKPAAETPQQRLWNSNVDLVVPNFHTPSVYFYRPTGSPNFFDGKVLKEALSKALVPFYPMAGRLCRDEDGRIEIDCKGQGVLFVEAESDGVVDDFGDFAPTLELRQLIPAVDYSQGIQSYALLVLQITHFKCGGVSLGVGMQHHAADGASGLHFINTWSDMARGLDLTIPPFIDRTLLRARDPPQPQFPHVEYQPPPTLKVTPENTPISEAVPETSVSIFKLTRDQINTLKAKSKEDGNTVNYSSYEMLAGHVWRSTCMARGLAHDQETKLYIATDGRSRLRPSLPPGYFGNVIFTTTPIAVAGDIQSKPIWYAASKLHDALARMDNDYLRSALDYLELQPDLKALVRGAHTFKCPNLGITSWSRLPIHDADFGWGRPIFMGPGGIAYEGLSFILPSPTNDGSQSVAISLQAEHMKLFEKFLYDF >Nt_cl_5b_CAE46932.1MGSEKMMKINIKESTLVKPSKPTPTKRLWSSNLDLIVGRIHLLTVYFYKPNGSSNFFDSKIMKEALSNVLVSFYPMAGRLARDEQGRIEINCNGEGVLFVEAESDAFVDDFGDFTPSLELRKLIPTVDTSGDISTFPLIIFQVTRFKCGGVSLGGGVFHTLSDGLSSIHFINTWSDIARGLSVAIPPFIDRTLLRARDPPTSSFEHVEYHPPPSLISSSKSLESTSPKPSTTTMLKFSSDQLGLLKSKSKHDGSTYEILAAHIWRCTCKARALSDDQLTKLHVATDGRSRLCPPLPPGYLGNVVFTGTPMAKSSELLQEPLTNSAKRIHSALSKMDDNYLRSALDYLELLPDLSALIRGPTYFASPNLNINSWTRLPVHDSDFGWGRPIHMGPACILYEGTVYILPSPNSKDRNLRLAVCLDADHMPLFEKYLYEF
