# Supplementary figures and images for: Genome-Wide Identification of BAHD Acyltransferases and In vivo Characterization of HQT-like Enzymes Involved in Caffeoylquinic Acid Synthesis in Globe Artichoke
Source: Front Plant Sci. 2016 Sep 23;7:1424. doi: 10.3389/fpls.2016.01424 (PMC5033976; doi:10.3389/fpls.2016.01424)

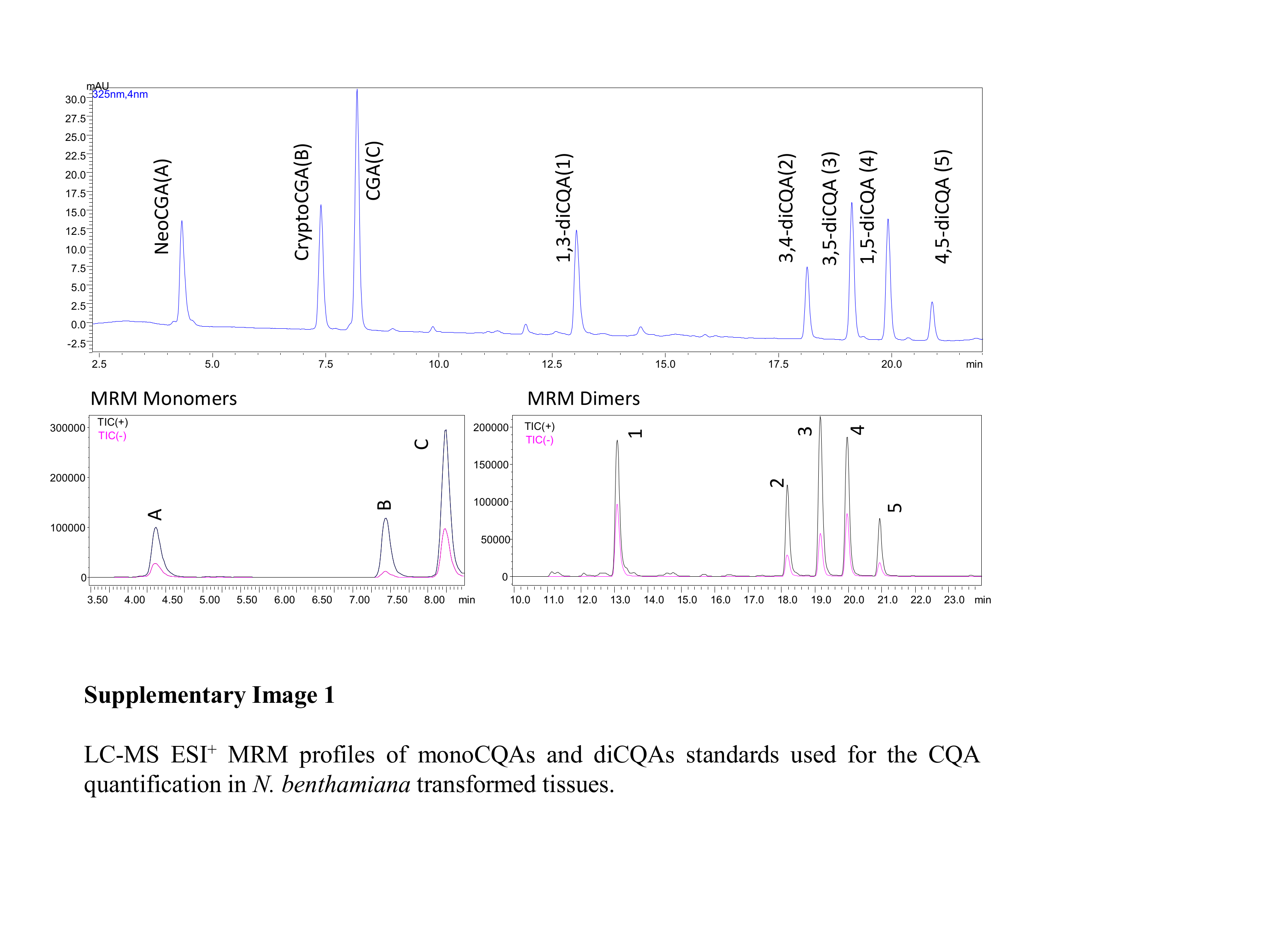

Supplement: Supplementary file 3 [file Image_1.TIFF]

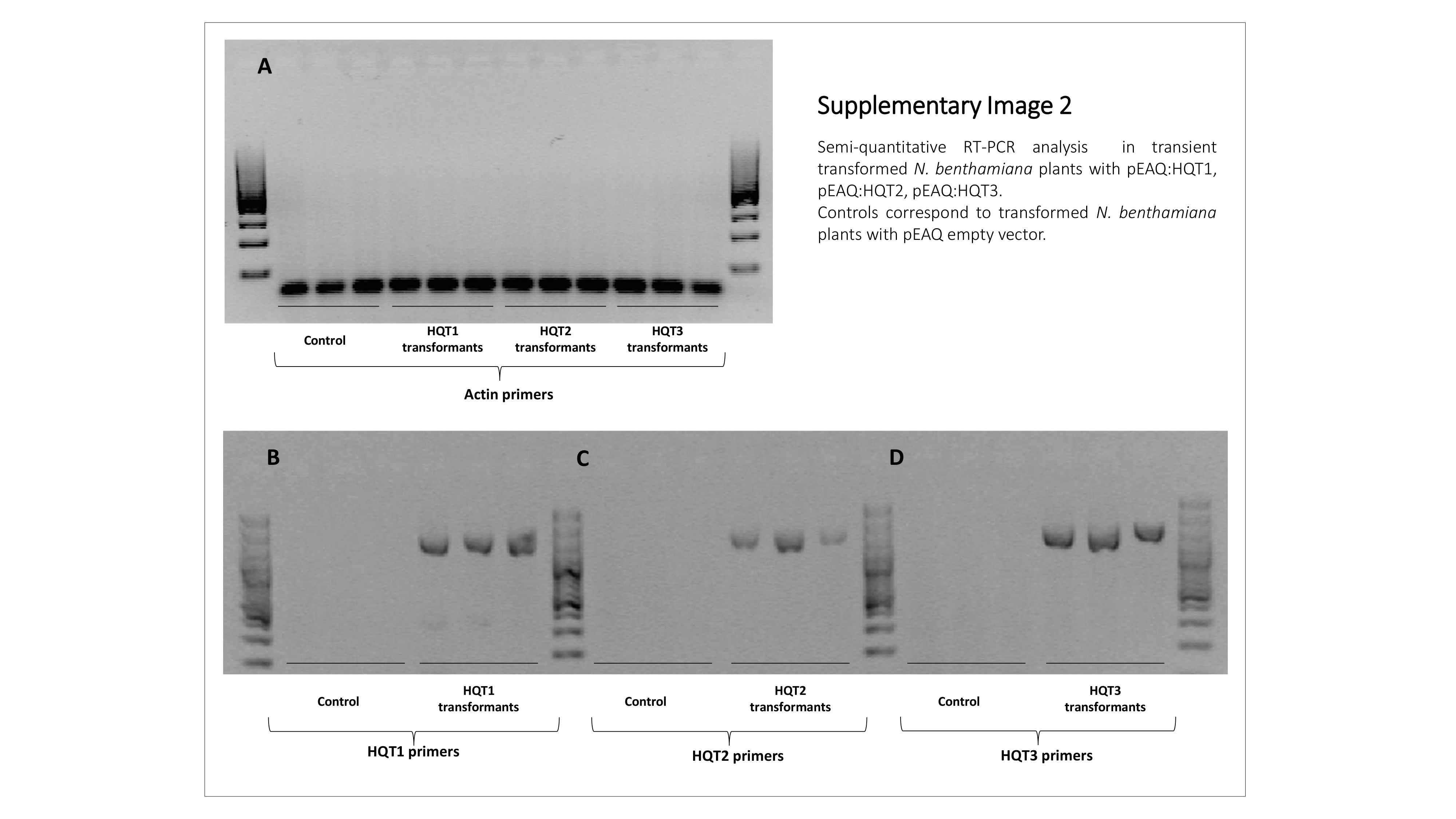

Supplement: Supplementary file 4 [file Image_2.TIFF]

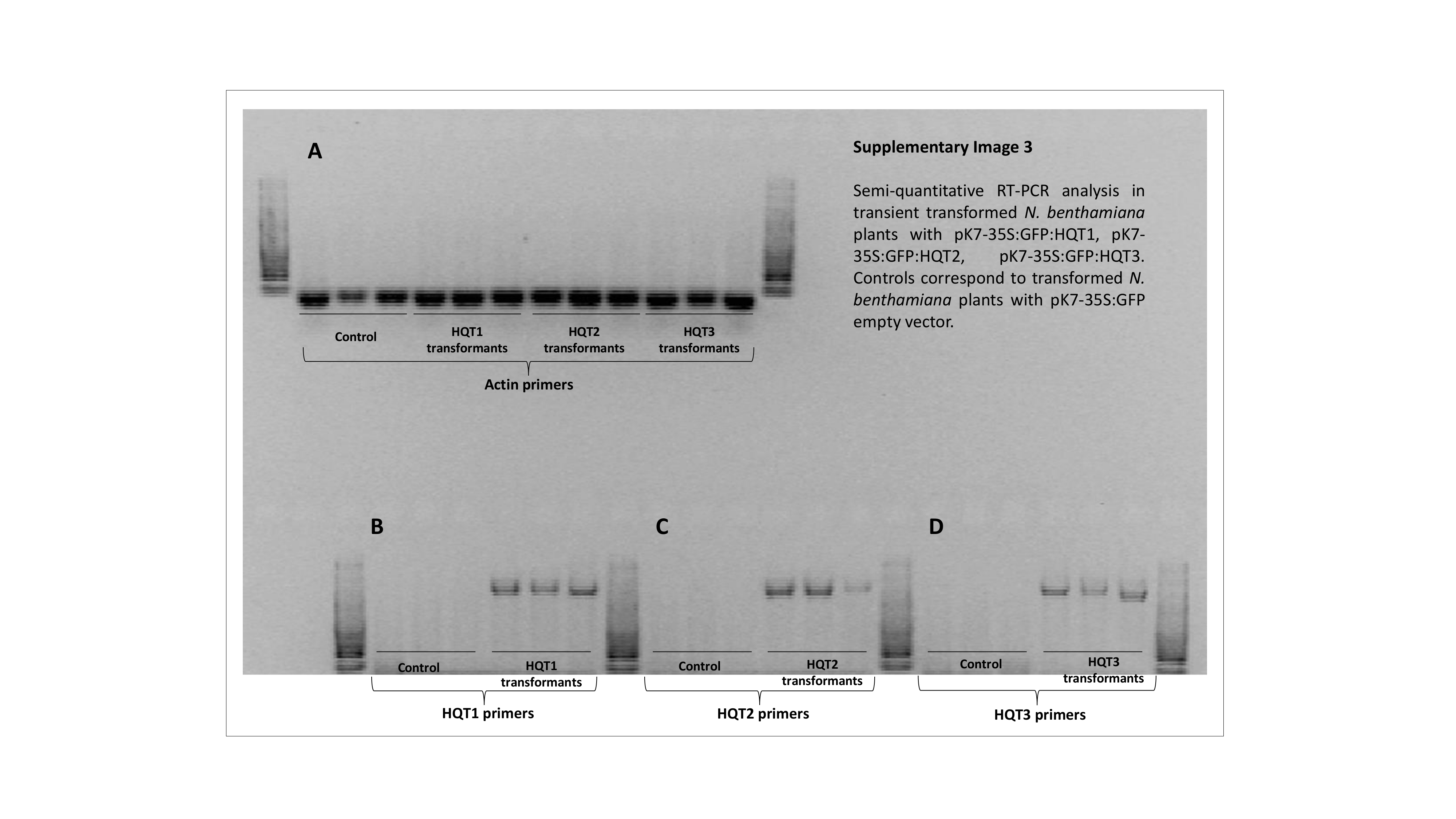

Supplement: Supplementary file 5 [file Image_3.TIFF]
